# Supplementary material for: Toxicology Study of Intra-Cisterna Magna Adeno-Associated Virus 9 Expressing Human Alpha-L-Iduronidase in Rhesus Macaques
Source: Mol Ther Methods Clin Dev. 2018 Jul 14;10:79–88. doi: 10.1016/j.omtm.2018.06.003 (PMC6070681; doi:10.1016/j.omtm.2018.06.003)
Supplement: Document S1. Figure S1 and Tables S1–S7 [file mmc1.pdf]

OMTM, Volume *10*

Supplemental Information

Toxicology Study of Intra-Cisterna Magna Adeno-Associated Virus 9 Expressing Human Alpha-L-Iduronidase in Rhesus Macaques

Juliette Hordeaux, Christian Hinderer, Tamara Goode, Nathan Katz, Elizabeth L. Buza, Peter Bell, Roberto Calcedo, Laura K. Richman, and James M. Wilson

Figure S1. Blood anomalies induced by the IS regimen. WBC counts, neutrophils, hemoglobin, ALT, phosphate, and albumin levels were transiently modified in at least one IS animal, after the onset of IS and before vector dosing, without any relation to intrathecal AAV9.hIDUA administration. Dotted lines represent the baseline average plus or minus two standard deviations. Black = vehicle control; red = HD; green = LD; purple = HD IS.

Figure S1.

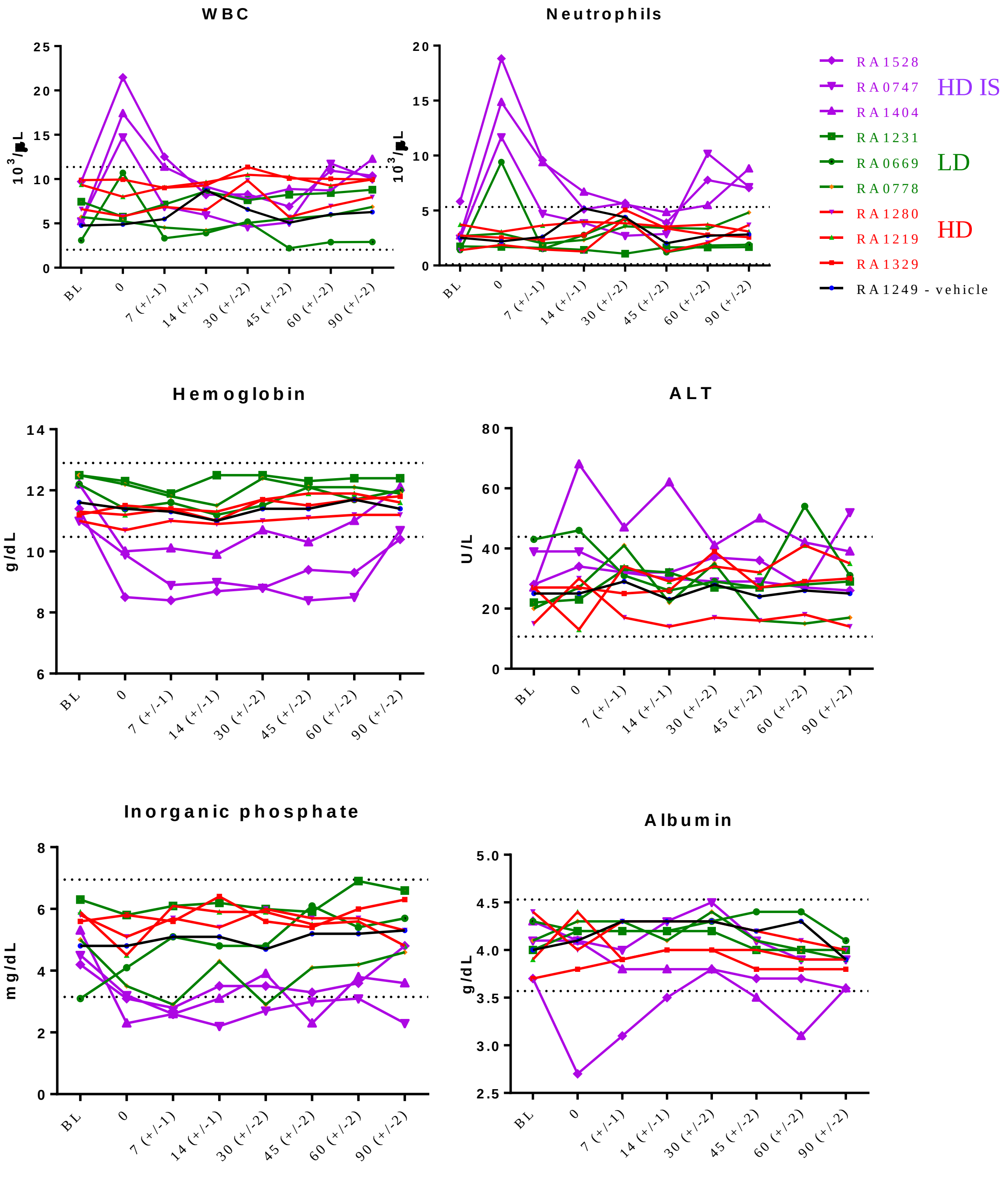
WBC
Neutrophils
Hemoglobin
ALT
Inorganic phosphate
Albumin
$10^3/\mu L$
g/dL
U/L
mg/dL
BL
0
7 (+/-1)
14 (+/-1)
30 (+/-2)
45 (+/-2)
60 (+/-2)
90 (+/-2)
RA1528
RA0747
RA1404
HD IS
RA1231
RA0669
RA0778
LD
RA1280
RA1219
RA1329
HD
RA1249 - vehicle

Table S1. Animal weights, study RGX150210p

Animal ID	Group		BL	D0	D3	D7 (+/-2)	D14 (+/-2)	D21 (+/-2)	D30 (+/-2)	D45 (+/-2)	D60 (+/-2)	D90 (+/-2)	D120 (+/-2)	D150 (+/-2)	D180 (+/-2)
RA1492	1A	HD D14	4.51	4.49	4.5	4.58	4.7	NA	NA	NA	NA	NA	NA	NA	NA
RA1292			5.49	5.54	5.66	5.48	5.51	NA	NA	NA	NA	NA	NA	NA	NA
RA0502			5.19	5.22	5.26	5.34	5.46	NA	NA	NA	NA	NA	NA	NA	NA
RA1470	1B	HD D90	4.93	5.05	5.05	4.92	5	5.27	5.6	5.91	5.75	5.9	NA	NA	NA
RA1514			4.41	4.33	4.39	4.4	4.64	4.41	4.39	4.86	4.95	4.65	NA	NA	NA
RA1287			5.36	5.44	5.49	5.66	5.55	5.71	5.6	5.55	5.7	5.75	NA	NA	NA
RA0773	1C	HD D180	5.65	5.75	5.79	5.98	6.08	6.19	6.45	6.51	6.3	6.5	6.25	5.85	6.05
RA1304			5.25	5.25	5.25	5.18	5.26	5.39	5.14	5.61	5.65	5.7	6.25	6.45	6.65
RA1532			5.39	5.74	5.86	5.8	5.9	6.07	6	5.7	6.05	6.45	6.35	6.05	6.1
RA1534	2	vehicle	5.09	5.15	5.09	5.2	5.4	NA	NA	NA	NA	NA	NA	NA	NA
RA0775			5.84	5.85	5.78	5.72	5.93	6.17	6.1	6.6	7.5	6.4	NA	NA	NA
RA1314			5.45	5.44	5.44	5.52	5.64	5.79	5.79	5.95	6	6.1	NA	NA	NA

Table S2. Animal weights, study RGX160822p

Animal ID	Group		BL	D0	D7 (+/- 1)	D14 (+/- 1)	D30	D45 (+/- 1)	D60 (+/- 2)	D90 (+/- 3)
RA1249	1	vehicle	6.60	6.55	6.50	6.60	6.80	6.75	6.90	7.30
RA1329	2	HD	8.85	8.75	8.85	9.00	9.10	8.50	9.00	9.20
RA1219			6.90	6.80	7.00	7.05	7.20	7.10	7.15	7.45
RA1280			7.80	7.90	7.85	8.00	8.30	8.60	8.75	9.25
RA0778	3	LD	4.10	4.10	4.05	4.10	4.10	4.20	4.30	4.25
RA0669			5.80	5.80	5.80	5.90	6.45	6.15	6.15	6.40
RA1231			8.05	7.80	7.75	7.85	7.85	8.10	8.20	8.70
RA1528	4	HD + IS	5.70	5.55	5.40	5.50	5.30	5.60	5.45	5.50
RA0747			5.85	5.75	5.65	5.80	5.75	5.70	5.70	5.85
RA1404			5.60	5.30	5.25	5.30	5.30	5.35	5.25	5.10

Table S3. CSF WBC counts

	D0	D3	D7	D14	D21	D30	D45	D60	D90	D120	D150	D180
RA1534 - vehicle	0	0	0	0	N/A	N/A	N/A	N/A	N/A	N/A	N/A	N/A
RA0775 - vehicle	0	0	0	0	0	0		0	0	N/A	N/A	N/A
RA1314 - vehicle	Blood	1	0	1	0	4	0	0	0	N/A	N/A	N/A
RA1249 - vehicle	0	N/A	0	Blood	Blood	0	1	1	0	N/A	N/A	N/A
RA0778 LD	0	N/A	Blood	0	N/A	1	1	0	0	N/A	N/A	N/A
RA0669 LD	0	N/A	0	0	N/A	0	7	10		N/A	N/A	N/A
RA1231 LD	0	N/A	0	0	N/A	2	1	2	0	N/A	N/A	N/A
RA1492 HD	0	0	0	0	N/A	N/A	N/A	N/A	N/A	N/A	N/A	N/A
RA1292 HD	4	1	2	0	N/A	N/A	N/A	N/A	N/A	N/A	N/A	N/A
RA0502 HD	0	1	0	0	N/A	N/A	N/A	N/A	N/A	N/A	N/A	N/A
RA1470 HD	Blood	2	0	17	12	Blood	28	14	4	N/A	N/A	N/A
RA1514 HD	Blood	3	0	0	12	11	4	1	1	N/A	N/A	N/A
RA1287 HD	0	0	0	0	10	27	2	1	0	N/A	N/A	N/A
RA1329 HD	0	N/A	0	0	N/A	0	0	4	2	N/A	N/A	N/A
RA1219 HD	0	N/A	0	0	N/A	*10.0**	1	2	0	N/A	N/A	N/A
RA1280 HD	0	N/A	0	0	N/A	3	2	2	0	N/A	N/A	N/A
RA0773 HD	0	0	0	0	4	4	11	1	0	1	0	0
RA1304 HD	0	1	3	0	1	1	0	2	1	3	0	2
RA1532 HD	0	1	0	6	18	27	11	3	7	16	13	Blood
RA1404 HD + IS	0	N/A	1	1	N/A	0	0	0	0	N/A	N/A	N/A
RA1528 HD + IS		N/A	0	0	N/A	0	0	0	0	N/A	N/A	N/A
RA0747 HD + IS	1	N/A	1	*10.00**	N/A	0	0	0	0	N/A	N/A	N/A

** values excluded: not confirmed by cytosmear analysis*

Blood: values excluded when red blood cell count > 500

Table S4. PBMC IFN-ϒ ELISPOT

			Average SFU per million PBMC							
**Group**	**Animal**	**Study Day**	**Medium**	AAV9-A	AAV9-B	AAV9-C	hIDUA-A	hIDUA-B	hIDUA-C	PHA + ctrl
Vehicle D14	RA1534	0	23	13	23	8	23	0	18	1490
		14	23	28	10	13	13	10	28	**1350**
HD D14	RA1492	0	20	20	18	13	23	15	15	**1330**
		14	25	13	23	10	5	20	15	**663**
	RA1292	0	23	33	13	18	15	8	15	**608**
		14	25	13	8	0	18	8	18	**515**
	RA0502	0	15	8	13	10	5	13	3	**570**
		14	15	18	8	8	15	13	5	**980**
LD D90	RA0778	0	8	28	20	53	40	23	25	**TNTC**
		14	18	43	**60**	**60**	**168**	**88**	53	**TNTC**
		30	25	25	23	23	**205**	NR	NR	**TNTC**
		60	80	65	110	105	**313**	233	**358**	**TNTC**
		90	28	33	50	28	**120**	**93**	**115**	**TNTC**
	RA0669	0	0	5	0	5	15	5	0	**1235**
		14	5	15	0	0	5	5	10	**TNTC**
		30	5	0	3	0	NR	NR	NR	**TNTC**
		60	13	18	**105**	**155**	**150**	**215**	**83**	**TNTC**
		90	0	15	23	**80**	45	**80**	10	**TNTC**
	RA1231	0	3	3	5	3	**63**	3	8	**TNTC**
		14	8	0	18	10	**85**	8	3	**TNTC**
		30	13	0	13	3	**120**	33	20	**TNTC**
		60	13	10	8	15	**113**	**128**	25	**TNTC**
		90	3	0	0	5	28	10	13	**TNTC**
HD D90	RA1470	0	5	5	5	0	8	5	23	**TNTC**
		21	5	8	5	15	8	3	3	**TNTC**
		90	3	15	40	8	**73**	8	10	**TNTC**
	RA1514	0	13	18	20	25	40	10	28	**TNTC**
		21	10	25	28	30	45	45	15	**TNTC**
		90	8	13	15	8	**100**	20	10	**1325**
	RA1287	0	5	10	8	5	5	30	10	**1185**
		21	5	8	10	25	53	25	8	**TNTC**
		90	5	15	13	20	50	**85**	8	**888**
	RA1329	0	0	3	13	0	3	8	3	**TNTC**
		14	3	35	20	5	**63**	23	15	**TNTC**
		30	3	**88**	3	25	**270**	**168**	48	**TNTC**
		60	25	18	35	8	**90**	53	23	**TNTC**
		90	8	20	5	0	43	20	18	**TNTC**
	RA1219	0	3	5	3	0	5	**108**	5	**TNTC**
		14	15	10	13	20	28	**138**	10	**TNTC**
		30	8	25	10	18	28	**73**	25	**TNTC**
		60	5	0	8	13	20	**58**	43	**TNTC**
		90	3	0	0	0	3	5	3	**TNTC**
	RA1280	0	13	15	20	10	30	10	13	**TNTC**
		14	20	8	20	10	48	5	13	**TNTC**
		30	25	10	20	25	**78**	45	35	**TNTC**
		60	20	5	20	18	**85**	35	60	**TNTC**
		90	3	18	10	20	55	15	23	**TNTC**
HD + IS D90	RA1404	0	5	10	5	5	5	0	35	**TNTC**
		30	0	0	0	0	3	3	3	**TNTC**
		60	0	0	3	3	0	5	5	**TNTC**
		90	35	13	20	40	28	25	20	**TNTC**
	RA1528	0	0	0	13	15	13	8	0	**TNTC**
		30	8	5	13	10	8	5	0	**TNTC**
		60	8	13	28	35	5	18	13	**TNTC**
		90	13	38	40	**85**	28	25	30	**TNTC**
	RA0747	0	5	13	18	8	18	15	8	**TNTC**
		30	0	13	10	8	20	0	5	**TNTC**
		60	20	23	33	38	13	20	0	**TNTC**
		90	23	33	68	**160**	**110**	**95**	38	**TNTC**

Table S4. (cont.) PBMC IFN-ϒ ELISPOT

			Average SFU per million PBMC							
**Group**	**Animal #**	**Study Day**	**Medium**	**AAV9 A**	**AAV9 B**	**AAV9 C**	**hIDUA A**	**hIDUA B**	**hIDUA C**	**PHA + ctrl**
Vehicle D90	RA0775	0	20	13	55	35	40	38	38	**TNTC**
		21	13	13	25	25	10	15	20	**TNTC**
		90	23	30	30	35	20	30	10	**TNTC**
	RA1314	0	23	13	13	5	18	18	10	**1340**
		21	13	3	10	10	0	15	3	**1390**
		90	3	18	13	13	33	10	20	**TNTC**
	RA1249	0	15	20	0	5	5	5	0	**1425**
		14	28	8	13	35	18	25	**125**	**TNTC**
		30	28	43	25	NR	25	20	NR	**TNTC**
		60	53	63	33	20	28	30	35	**TNTC**
		90	8	8	3	10	10	0	18	**TNTC**
HD D180	RA0773	0	5	15	3	10	10	5	15	**1520**
		21	3	8	3	10	13	10	0	**1560**
		90	8	0	8	5	10	15	8	**900**
		180	30	15	20	33	45	45	20	**2000**
	RA1304	0	8	0	0	0	0	0	0	**265**
		21	5	3	3	0	5	3	5	**95**
		90	3	3	8	8	0	13	8	**253**
		180	15	18	18	28	28	20	13	**503**
	RA1532	0	5	3	0	5	8	0	3	**TNTC**
		21	0	3	0	3	8	8	8	**TNTC**
		90	23	23	13	30	**70**	55	20	**TNTC**
		180	18	**68**	**65**	**108**	**95**	**63**	**65**	**TNTC**

SFU = Spot Forming Unit. NR = not enough cells

Positive responses, defined as an average value >55 SFU/million PBMCs and at least three times greater than the medium negative control, are highlighted in red. TNTC = too numerous to be counted

Table S5. Incidence and severity of the DRG finding

Study	RGX151002p					RGX160822p			
**Necropsy Day**	**14**		**90**		**180**	**90**			
**Group**	**1A**	**2A**	**1B**	**2B**	**1C**	**1**	**2**	**3**	**4**
**Dose (GC)**	**10^{13}**	**Control**	**10^{13}**	**Control**	**10^{13}**	**Control**	**10^{13}**	**10^{12}**	**10^{13}**
**Immunosuppression**	**No**	**No**	**No**	**No**	**No**	**No**	**No**	**No**	**Yes**
**Number of Animals**	**3**	**1**	**3**	**2**	**3**	**1**	**3**	**3**	**3**
No. examined	0	0	0	0	0	1	3	3	3
**Dorsal root ganglion, Cervical**									
Neuronal cell body degeneration with mononuclear cell infiltrate									
No significant abnormalities	-	-	-	-	-	1 (M)	1	1	1
Grade 1	-	-	-	-	-	-	2	2	2
**Dorsal root ganglion, Thoracic**									
Neuronal cell body degeneration with mononuclear cell infiltrate									
No significant abnormalities	-	-	-	-	-	1	0	1	1
Grade 1	-	-	-	-	-	-	3	2	2
**Dorsal root ganglion, Lumbar**									
Neuronal cell body degeneration with mononuclear cell infiltrate									
No significant abnormalities	-	-	-	-	-	1	0	0	2
Grade 1	-	-	-	-	-	-	3	3	-
Grade 2	-	-	-	-	-	-	-	-	1

M=missing tissue

The findings in the DRG consisted of neuronal degeneration characterized by central chromatolysis, satellitosis, and mononuclear cell infiltrates that surrounded and invaded neuronal cell bodies (neuronophagia). DRG from the initial study, RGX151002p, were not sampled.

Table S6. Incidence and severity of the axonopathy finding

**Study**	**RGX151002p**					**RGX160822p**			
**Necropsy Day**	**14**		**90**		**180**	**90**			
**Group**	**1A**	**2A**	**1B**	**2B**	**1C**	**1**	**2**	**3**	**4**
**Dose (GC)**	**10^{13}**	**Control**	**10^{13}**	**Control**	**10^{13}**	**Control**	**10^{13}**	**10^{12}**	**10^{13}**
**Immunosuppression**	**No**	**No**	**No**	**No**	**No**	**No**	**No**	**No**	**Yes**
**Number of Animals**	**3**	**1**	**3**	**2**	**3**	**1**	**3**	**3**	**3**
No. examined	3	1	3	2	3	1	3	3	3
**Spinal Cord, Cervical**									
Axonopathy, dorsal white matter tracts									
No significant abnormalities	3	1	0	2	0	1	1	0	2
Grade 1	-	-	-	-	2	-	-	2	1
Grade 2	-	-	2	-	1	-	2	1	-
Grade 3	-	-	1	-	-	-	-	-	-
**Spinal Cord, Thoracic**									
Axonopathy, dorsal white matter tracts									
No significant abnormalities	3	1	0	2	0	1	0	0	1
Grade 1	-	-	2	-	2	-	2	3	2
Grade 2	-	-	1	-	1	-	1	-	-
**Spinal Cord, Lumbar**									
Axonopathy, dorsal white matter tracts									
No significant abnormalities	3	1	0	2	0	1	0	0	1
Grade 1	-	-	-	-	1	-	2	1	1
Grade 2	-	-	3	-	2	-	1	2	1

The dorsal white matter tracts of the spinal cord had an axonopathy characterized by dilated myelin sheaths with and without myelomacrophages, which was consistent with axonal degeneration.

Table S7. Biodistribution (vector GCs) in tissues

	HD D14			HD D90						HD D180			HD D90 + IS			LD		
	RA0502	RA1292	RA1492	RA1287	RA1470	RA1514	RA1219	RA1280	RA1329	RA0773	RA1304	RA1532	RA0747	RA1404	RA1528	RA0669	RA0778	RA1231
Frontal cortex	185000	33500	17600	17300	78500	9920	160000	24800	530	124000	61800	23500	48700	135000	40600	1770	11100	<50
Parietal cortex	30600	2700	3380	24700	33500	2780	26500	4660	595	44300	76000	50000	10800	154000	21800	181	3720	1520
Temporal cortex	29500	60600	16000	52200	215000	14900	368000	201000	137	34000	302000	23400	52700	78300	82600	2650	6900	2100
Occipital cortex	12200	9440	6490	34100	303000	6780	22500	23700	536	64500	4530	19900	21100	52600	8630	3280	2380	353
Hippocampus	3370	48400	4940	4110	94700	18100	23700	267000	97.6	6050	1400	49000	103000	13600	33800	15500	39200	3090
Medulla	5360	239	27700	32900	14100	19000	25000	250000	438	9170	82000	49900	18800	2800	7280	544	23200	846
Cerebellum	2390	2950	2660	300	8740	11800	6350	4690	368	67700	7960	6370	12900	11800	3550	387	1290	145
Cervical Spinal cord	39100	42800	7830	22200	35100	5370	62800	15700	561	28800	39900	23500	28400	61900	74300	5330	11500	1070
Thoracic spinal cord	80500	20500	14000	9460	2950	26900	22200	31800	785	22300	161000	11100	120000	77100	72200	7320	3890	4370
Lumbar Spinal cord	81400	39400	6590	4950	3080	12100	37800	34800	851	83700	38300	37400	79800	59900	135000	4970	26800	2490
Cervical DRG	N/A	N/A	N/A	N/A	N/A	N/A	208000	121000	N/A	N/A	N/A	N/A	209000	19600	125000	12600	32400	1990
Thoracic DRG	N/A	N/A	N/A	N/A	N/A	N/A	66600	47300	N/A	N/A	N/A	N/A	103000	66600	282000	1260	4310	232
Lumbar DRG	N/A	N/A	N/A	N/A	N/A	N/A	105000	43600	N/A	N/A	N/A	N/A	81800	121000	345000	3260	37300	1200
Eye	<50	int	237	301	17700	<50	N/A	N/A	N/A	105	130	82	N/A	N/A	N/A	N/A	N/A	N/A
Liver, Caudate lobe	415000	1070000	784000	1530000	181000	91100	N/A	N/A	N/A	1210000	763000	210000	N/A	N/A	N/A	N/A	N/A	N/A
Liver, Left lobe	307000	190000	519000	1350000	189000	167000	N/A	N/A	N/A	1380000	334000	110000	N/A	N/A	N/A	N/A	N/A	N/A
Liver, middle lobe	361000	159000	45300	1680000	324000	290000	N/A	N/A	N/A	1680000	268000	449000	N/A	N/A	N/A	N/A	N/A	N/A
Liver, right lobe	499000	250000	86900	135000	228000	114000	N/A	N/A	N/A	1040000	309000	110000	N/A	N/A	N/A	N/A	N/A	N/A
Left kidney	2570	1270	4130	5210	5000	945	N/A	N/A	N/A	882000	1580	430	N/A	N/A	N/A	N/A	N/A	N/A
Right kidney	3300	613	4510	9700	9280	870	N/A	N/A	N/A	775	3530	380	N/A	N/A	N/A	N/A	N/A	N/A
Spleen	536000	78200	301000	1360	7120	2140	N/A	N/A	N/A	154000	5860	23000	N/A	N/A	N/A	N/A	N/A	N/A
Heart	7430	11500	1820	1970	9670	2050	N/A	N/A	N/A	2400	5420	280	N/A	N/A	N/A	N/A	N/A	N/A
Lung, left	113	181	878	197	4270	438	N/A	N/A	N/A	145	556	int	N/A	N/A	N/A	N/A	N/A	N/A
Lung, right	211	1390	1440	148	1160	227	N/A	N/A	N/A	54.6	371	341	N/A	N/A	N/A	N/A	N/A	N/A
Thyroid	int	54.8	991	417	180	2750	N/A	N/A	N/A	831	84.9	63.3	N/A	N/A	N/A	N/A	N/A	N/A
Lymph node, axillary	61300	7550	int	5090	1310	4330	N/A	N/A	N/A	17200	11500	64500	N/A	N/A	N/A	N/A	N/A	N/A
Lymph node, inguinal	19500	16300	22200	7840	1450	1470	N/A	N/A	N/A	29700	13400	12000	N/A	N/A	N/A	N/A	N/A	N/A
Lymph node, mesenteri	12100	37400	18300	1590	2150	4030	N/A	N/A	N/A	9920	15500	9010	N/A	N/A	N/A	N/A	N/A	N/A
Lymph node, popliteal	113	15000	200	5540	27600	64.8	N/A	N/A	N/A	8650	1150	7100	N/A	N/A	N/A	N/A	N/A	N/A
Ovary, left	625	N/A	2440	N/A	3770	121	N/A	N/A	N/A	237	N/A	190	N/A	N/A	N/A	N/A	N/A	N/A
Ovary, right	555	N/A	881	N/A	127		N/A	N/A	N/A	1080	N/A	72.2	N/A	N/A	N/A	N/A	N/A	N/A
Testicle, left	N/A	2950	N/A	107	N/A	N/A	N/A	N/A	N/A	N/A	163	N/A	N/A	N/A	N/A	N/A	N/A	N/A
Testicle, right	N/A	888	N/A	262	N/A	N/A	N/A	N/A	N/A	N/A	144	N/A	N/A	N/A	N/A	N/A	N/A	N/A
Bone marrow	6270	14700	12500	734	4080	4410	N/A	N/A	N/A	11500	10600	7760	N/A	N/A	N/A	N/A	N/A	N/A
N/A Not Applicable																		
int. interference																		
