# Supplementary material for: Toxicology Study of Intra-Cisterna Magna Adeno-Associated Virus 9 Expressing Human Alpha-L-Iduronidase in Rhesus Macaques
Source: Mol Ther Methods Clin Dev. 2018 Jul 14;10:79–88. doi: 10.1016/j.omtm.2018.06.003 (PMC6070681; doi:10.1016/j.omtm.2018.06.003)
Supplement: Document S2. Article plus Supplemental Information [file mmc2.pdf]

Molecular Therapy
Methods & Clinical Development
Original Article

Toxicology Study of Intra-Cisterna Magna Adeno-Associated Virus 9 Expressing Human Alpha-L-Iduronidase in Rhesus Macaques

Juliette Hordeaux,[1] Christian Hinderer,[1] Tamara Goode,[1] Nathan Katz,[1] Elizabeth L. Buza,[1] Peter Bell,[1] Roberto Calcedo,[1] Laura K. Richman,[1] and James M. Wilson[1]

[1]Gene Therapy Program, Department of Medicine, University of Pennsylvania, Perelman School of Medicine, Philadelphia, PA 19104, USA

Mucopolysaccharidosis type I is a recessive genetic disease caused by deficiency of the lysosomal enzyme α-L-iduronidase, which leads to a neurodegenerative and systemic disease called Hurler syndrome in its most severe form. Several clinical trials are evaluating adeno-associated virus serotype 9 (AAV9) for the treatment of neurodegenerative diseases. Although these trials focus on systemic or lumbar administration, intrathecal administration via suboccipital puncture into the cisterna magna has demonstrated remarkable efficacy in large animals. We, therefore, conducted a good laboratory practice-compliant non-clinical study to investigate the safety of suboccipital AAV9 gene transfer of human α-L-iduronidase into nonhuman primates. We dosed 22 rhesus macaques, including three immunosuppressed animals, with vehicle or one of two doses of vector. We assessed in-life safety and immune responses. Animals were euthanized 14, 90, or 180 days post-vector administration and evaluated for histopathology and biodistribution. No procedure-related lesions or adverse events occurred. All vector-treated animals showed a dose-dependent mononuclear pleocytosis in the cerebrospinal fluid and minimal to moderate asymptomatic degeneration of dorsal root ganglia neurons and associated axons. These studies support the clinical development of suboccipital AAV delivery for Hurler syndrome and highlight a potential sensory neuron toxicity that warrants careful monitoring in first-in-human studies.

INTRODUCTION

Mucopolysaccharidosis type I (MPS I) is a rare inherited disorder caused by mutations in the gene encoding α-L-iduronidase (IDUA), a lysosomal enzyme required for degradation of glycosaminoglycans, including heparan sulfate and dermatan sulfate. IDUA deficiency results in accumulation of these glycosaminoglycan substrates in cells throughout the body, leading to a diverse array of clinical manifestations. Hepatosplenomegaly, bone deformities, corneal clouding, and cardiac valve stenosis or insufficiency are common complications.[1,2] Patients with mutations that yield little or no residual expression of active IDUA present with a severe form of the disease, termed Hurler syndrome. In addition to the somatic manifestations of the disease described above, Hurler syndrome is characterized by marked lysosomal storage lesions in the CNS and progressive cognitive impairment beginning in early childhood.[1,3]

Two treatment options are currently available for MPS I. Both are based on the discovery of mannose-6-phosphate receptor-mediated transport of lysosomal enzymes, which allows for extracellular IDUA to undergo endocytosis and trafficking to the lysosome.[4,5] Intravenous enzyme replacement therapy can deliver the enzyme to most tissues and improves many of the systemic manifestations of the disease.[6–8] However, intravenous delivery of IDUA does not achieve significant CNS penetration and does not attenuate cognitive decline in the severe Hurler syndrome.[9] In contrast, hematopoietic stem cell transplantation can achieve enzyme delivery to the CNS, as some donor-derived macrophage progenitor cells migrate into the brain, where they secrete IDUA.[10] However, stem cell transplantation is associated with morbidity and mortality, and typically accomplishes incomplete preservation of cognitive function, likely because of limited enzyme delivery to the brain and the slow kinetics of donor cell engraftment in the CNS.[11]

With the goal of safely and rapidly reconstituting therapeutic levels of IDUA activity in the CNS, we developed an approach in which the IDUA coding sequence expressed from a strong ubiquitous promoter is delivered to cells throughout the CNS using an adeno-associated virus (AAV) vector injected into the cerebrospinal fluid (CSF). Preclinical studies in naturally occurring canine and feline models of MPS I demonstrated that a single minimally invasive injection of an AAV serotype 9 (AAV9) vector into the cisterna magna (ICM) can achieve high levels of IDUA activity in the CSF within a week of administration, leading to the resolution of storage lesions throughout the brain.[12–14] The ICM approach results in superior CNS distribution of AAV vectors compared with other routes of CSF access but is not commonly utilized in clinical practice,[15]

Received 6 April 2018; accepted 7 June 2018;
https://doi.org/10.1016/j.omtm.2018.06.003.

Correspondence: James M. Wilson, MD, PhD, Gene Therapy Program, University of Pennsylvania, Perelman School of Medicine, 125 South 31st Street, Suite 1200, Philadelphia, PA 19104, USA.
E-mail: wilsonjm@upenn.edu

This is an open access article under the CC BY license (http://creativecommons.org/licenses/by/4.0/).

Table 1. Study Design

Study	RGX151002p					RGX160822p			
Necropsy Day	14		90		180	90			
Group	1A	2A	1B	2B	1C	1	2	3	4
Dose (GC)	10^{13}	control	10^{13}	control	10^{13}	control	10^{13}	10^{12}	10^{13}
Immunosuppression	no	no	no	no	no	no	no	no	yes
No. of animals	3	1	3	2	3	1	3	3	3

Study RGX151002p was conducted first. RGX160822p was conducted later in order to further investigate findings from RGX151002p.

necessitating the evaluation of the safety of the procedure in preclinical studies.

In order to advance this approach to the clinic, we performed a good laboratory practice-compliant toxicology study in adult rhesus macaques. The use of nonhuman primates was essential for this study, as the size and anatomy of these animals enabled us to utilize the same image-guided ICM delivery approach that will be employed in clinical studies. Furthermore, nonhuman primates better recapitulate host innate and adaptive immune responses to vector and transgene-derived IDUA that are likely to be seen in humans. The size and anatomic similarity of nonhuman primates to humans also make them a relevant model to evaluate vector biodistribution after ICM injection and any toxicity associated with vector delivery to target cells.

RESULTS

Study Design

In this study, we utilized two experimental designs to evaluate the safety, biodistribution, and pharmacology of a vector encoding human IDUA (hIDUA) for up to 180 days after administration by image-guided ICM suboccipital puncture in rhesus macaques (see Table 1 for study design). We evaluated a high dose (HD) of 1×10^{13} genome copies (GCs; n = 15) at three different time points (14, 90, and 180 days postinjection) and a low dose (LD) of 1×10^{12} GCs (n = 3; 90 days postinjection) compared with vehicle control (n = 4). Three HD subjects from the 90-day cohort also received immunosuppression (IS) consisting of mycophenolate mofetil (MMF) and rapamycin (Table 2).

In-Life Safety Parameters

We performed sample collection and necropsies at the time points indicated in Table 2. No adverse events occurred during the administration procedure, and all animals recovered uneventfully from anesthesia. Animals from both studies survived until their scheduled necropsy time point at days 14, 90, or 180 post-vector administration. We did not observe any abnormalities during the course of the study in animals that received the test article in terms of cage-side observations, temperature, heart rate, or respiratory rate (recorded in sedated animals).

The IS regimen administered to three animals caused adverse effects of decreased appetite, diarrhea, and weight loss that all started prior to test article administration. Most affected animals had culture or PCR testing of stools positive for *Campylobacter* spp. and *Helicobacter* spp. We provided symptomatic and supportive care, as well as antibiotic treatments (trimethoprim and sulfamethoxazole, erythromycin, or enrofloxacin based on cultures and response to treatment), to control the gastrointestinal symptoms. One animal (RA1404) did not respond to treatment; therefore, the study veterinarian decided to stop MMF on day 35, leading to complete resolution of the symptoms.

All non-IS animals maintained normal body weights throughout the studies (Tables S1 and S2). Two IS animals (RA1528 and RA1404) started losing weight soon after the IS regimen was started, before vector dosing. We attributed the cause to repeated episodes of diarrhea and decreased appetite either directly related to the IS drugs, the daily orogastric tubing procedure used to administer the IS drugs, opportunistic enteric pathogens, or a combination of the above.

Pathology

Pathology consisted of complete blood cell (CBC) count, blood chemistry and electrolyte panels, a coagulation panel (thromboplastin time, activated partial thromboplastin time, fibrinogen, D-dimer, and fibrin degradation products), CSF white blood cell (WBC) count, glucose and protein levels, and cytosmear analysis (Figure S1 and data not shown). We have presented only the parameters with test article-related abnormalities, defined as modifications that (1) exceeded the baseline average ±2 SDs; (2) occurred after administering the test article; and (3) were not observed in the control group. Among non-IS animals, we did not find any significant abnormalities in the CBC, blood chemistry (including transaminases), or coagulation parameters (data not shown); all parameters were comparable with control and/or baseline average ±2 SDs and/or within the range of normal variability for this species. The three IS animals exhibited changes in the CBC count after the onset of the IS regimen (Figure S1). These changes initially emerged before vector dosing and were thus not related to the test article. Anomalies present in the three IS animals were a transient neutrophilic leukocytosis on day 0 and an anemia that resolved after MMF withdrawal on day 60. Blood chemistry was normal for the most part, except for a minimal increase in alanine aminotransferase (2.5-fold increase relative to baseline) in animal RA1404. This increase returned to baseline levels by day 90. All IS animals had low inorganic phosphate values, and two IS animals had

Table 2. Animals and Study Dates

Group	Animal No.	Gender	Weight (kg)	Vector Dose	IS Regimen	Blood + CSF Analysis	Necropsy
Vehicle D14	RA1534	F	5.09	–	–		
	RA1492	F	4.51		–		
HD D14	RA1292	M	5.49	1 × 10^{13} GCs	–	D0, D3, D7, D14	D14
	RA0502	F	5.19		–		
	RA0775	F	5.84		–		
Vehicle D90	RA1314	M	5.45	–	–	D0, D3, D7, D14, D21,[a] D30, D45, D60, D90	
	RA1249	M	6.60		–		
	RA0778	F	4.10		–		
LD D90	RA0669	M	5.80	1 × 10^{12} GCs	–	D0, D7, D14, D21,[a] D30, D45, D60, D90	
	RA1231	M	8.05		–		
	RA1470	F	4.93		–		
	RA1514	F	4.41		–	D0, D3, D7, D14, D21,[a] D30, D45, D60, D90	D90
HD D90	RA1287	M	5.36		–		
	RA1329	M	8.85		–		
	RA1219	M	6.90	1 × 10^{13} GCs	–		
	RA1280	M	7.80		–	D0, D7, D14, D21,[a] D30, D45, D60, D90	
	RA1404	M	5.60				
HD + IS D90	RA1528	F	5.70		rapamycin + MMF: up to D60[b] rapamycin: D60–D90		
	RA0747	F	5.85				
	RA0773	F	5.65		–		
HD D180	RA1304	M	5.25	1 × 10^{13} GCs	–	D0, D3, D7, D14, D21,[a] D30, D45, D60, D90, D120, D150, D180	D180
	RA1532	F	5.39		–		

[a]D21 was not sampled for study RGX160822p.
[b]RA1404 MMF was stopped on day 35 because of recurrent diarrhea and weight loss.

low albumin levels. We believe these levels were caused by frequent diarrhea, which can lead to intestinal malabsorption.

CSF analysis revealed treatment-related changes. A mild mononuclear pleocytosis (5–30 cells/μL) occurred in most HD-treated animals and in one LD-treated animal (Figures 1A–1D; Table S3). The IS and vehicle control animals had normal CSF parameters throughout the study. The pleocytosis (mainly lymphocytes with fewer macrophages) appeared as early as day 14, peaked between days 21 and 45, and typically resolved by day 90. Only one animal had a second peak of pleocytosis at day 120. In some animals, the elevated CSF nucleated cells were inconsistently paralleled by a mild transient increase in CSF protein. We did not observe any changes in CSF glucose concentration.

Immune Responses to hIDUA and AAV9 Capsid

We evaluated serum and CSF samples for antibodies against hIDUA using an ELISA (Figure 2A). Antibodies against hIDUA were detectable in the serum (dilution 1:1,000) and CSF (1:20) of all vector-treated animals that were not immune suppressed. The response was of similar intensity among LD and HD animals. IS completely ablated the formation of antibodies to hIDUA at the tested dilutions during the 90-day in-life phase of the study. Neutralizing antibodies (NAbs) to AAV9 emerged at levels that were similar across both non-IS dose cohorts. Peak titers reached 1:1,280–5,120 in the serum and 1:20–160 in the CSF of most animals (Figure 2B). IS markedly reduced the formation of AAV9 NAbs in serum, with levels ranging from 1:20 to 1:160.

We detected T cell responses to hIDUA peptide pools following vector administration in the peripheral blood mononuclear cells (PBMCs) of all HD and LD animals and in one of the three HD IS animals (Figure 2C; Table S4). T cell responses to AAV9 capsid occurred sporadically and with a low magnitude across all groups. T cell responses to the transgene product in the IS animals were less prevalent and occurred later compared with non-IS animals, likely reflecting MMF withdrawal on day 60. RA1404 had an earlier MMF withdrawal on day 35 and was maintained with rapamycin only until scheduled sacrifice. This animal did not exhibit any measurable T cell immune response.

Histopathology

Peripheral organs showed no gross or microscopic test article-related changes. Histologic findings related to the test article were visible in the dorsal root ganglia (DRG), the corresponding axons from dorsal

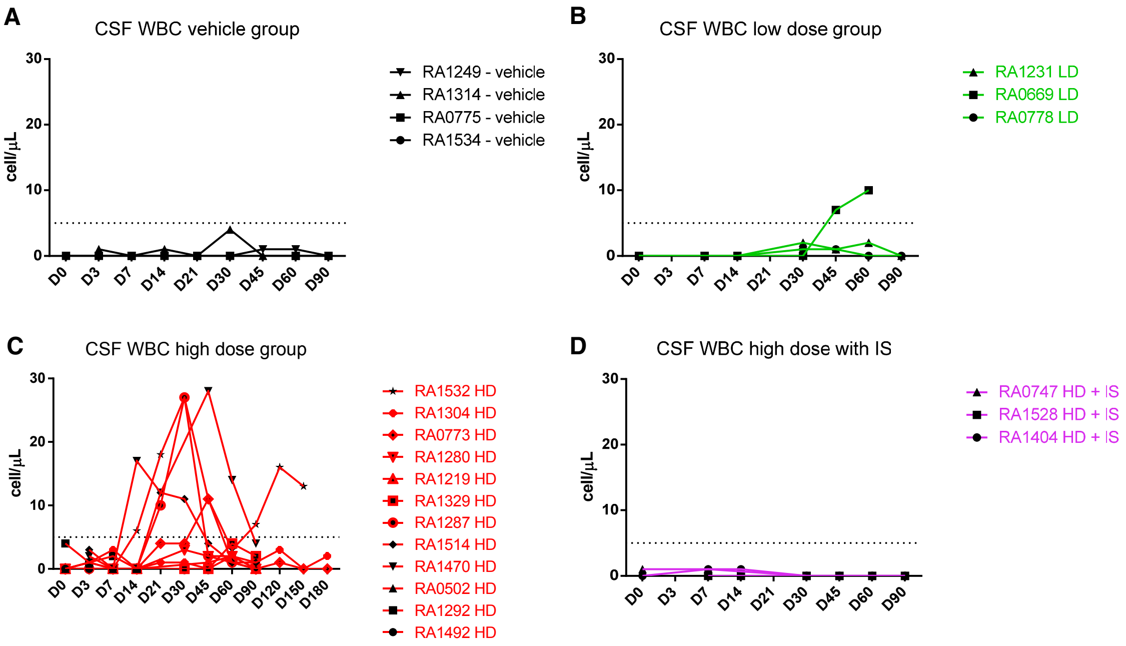

Figure 1. CSF WBC Counts in Rhesus Macaques Injected with Suboccipital AAV9.hIDUA

(A–D) WBC counts are shown for the (A) vehicle control group dosed with artificial CSF, (B) LD group dosed with 1×10^{12} GCs of AAV9.hIDUA, (C) HD group dosed with 1×10^{13} GCs of AAV9.hIDUA, and (D) HD IS group dosed with 1×10^{13} GCs of AAV9.hIDUA with IS (rapamycin and MMF with withdrawal of MMF on day 60). A dose-dependent minimal to mild CSF mononuclear pleocytosis (mainly lymphocytic) was observed in one LD, five HD, and no HD IS animals. Dashed lines indicate the threshold for abnormal CSF cell counts in rhesus macaques (5 cells/μL of CSF). Values were excluded when a blood contamination above 500 erythrocytes/μL was seen.

spinal cord white matter, and the trigeminal nerve ganglia. Tables S5 and S6 list and summarize the incidence and severity of the histopathology findings.

We observed minimal to mild neuronal cell body degeneration with mononuclear cell infiltration (Figures 3A and 3B) in at least one DRG and trigeminal nerve ganglia of all animals except one HD IS animal; note that the DRG and trigeminal nerve ganglia were collected only from animals in the second study. This finding was absent in the DRG of the vehicle control animal (Figure 3C). All spinal cord segments showed minimal to moderate axonopathy localized within the dorsal white matter tracts (i.e., axons projecting from DRG sensory neurons). This axonopathy was bilateral and characterized by dilated myelin sheaths with and without myelomacrophages, consistent with axonal degeneration (Figures 3D and 3E). Occasionally, axonal degeneration was also observed in the dorsal nerve roots of the spinal cord and, in one LD animal, in the sciatic nerve. This dorsal axonopathy was absent in the vehicle control (Figure 3F) but present in at least one spinal cord segment of all but one HD IS animal (Figure 3G). Overall, the incidence and severity of the DRG degeneration and axonopathy trended toward lower scores in the HD IS animals (Figure 3G). Additionally, we observed axonopathy in the spinal cord segment adjacent to the intrathecal injection site in all groups except the vehicle control group.

In the DRG, inflammatory cells were mostly $CD20^+$ B lymphocytes and $CD3^+$ T lymphocytes (Figure 3H). Overall, very few $CD68^+$ macrophages were present (data not shown). Lymphocytes were clustered around hIDUA-positive transduced neurons and sometimes formed small inflammatory nodules replacing missing neurons (neuronophagia). Nuclei that were not positive for lymphocyte or monocyte markers were likely satellite cells that were activated to proliferate.

A few animals showed minimal focal mononuclear cell infiltrates in the leptomeninges of the brain. The brain parenchyma itself (frontal, occipital, parietal, and temporal cortices, cerebellum, hippocampus, and medulla), as well as the spinal cord gray matter, were within normal limits in all animals.

Vector Biodistribution

We extracted DNA from tissues as listed in Table 2 and quantified the vector genomes. The complete biodistribution with individual results is detailed in Table S7. The average values per group are illustrated in Figure 4. We detected AAV vector genomes throughout the brain, spinal cord, and DRG of all vector-treated animals and found that levels in the HD group were approximately 10 times higher than the LD group. In the HD groups, GCs were about 1×10^5 GCs/μg DNA in the brain, spinal cord segments, and DRG without any notable differences between days 14, 90, and 180. Furthermore, we observed no discernable effect of the distance relative to the injection site in the spinal cord segments. Across all groups, the cerebellum contained the lowest copy numbers from the CNS. Vector GC levels

A

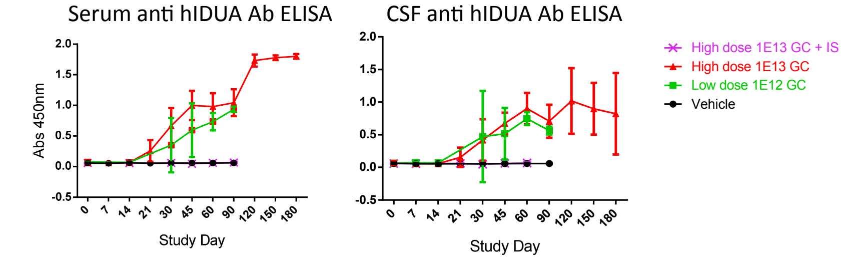

B

AAV9 neutralizing Ab titer

serum

	Animal ID	Day 0	Day 14	Day 30	Day 45	Day 60	Day 90
HD IS	RA1528	5	10	10	<5	5	160
	RA1404	<5	<5	<5	<5	<5	20
	RA0747	<5	10	5	<5	<5	80
HD	RA1329	<5	80	40	40	20	40
	RA1280	<5	160	80	1280	1280	1280
	RA1219	<5	80	320	640	2560	1280
LD	RA1231	<5	40	20	320	640	320
	RA0669	<5	80	320	320	1280	1280
	RA0778	<5	320	1280	640	5120	640
ctrl	RA1249	<5	<5	<5	5	5	<5

CSF

	Animal ID	Day 0	Day 7	Day 14	Day 30	Day 45	Day 60	Day 90
HD IS	RA1528	<5	<5	<5	<5	20	20	20
	RA1404	<5	<5	<5	<5	10	20	20
	RA0747	<5	<5	<5	<5	20	10	80
HD	RA1329	<5	<5	<5	<5	<5	<5	<5
	RA1280	<5	<5	<5	<5	160	40	<5
	RA1219	<5	<5	<5	<5	5	20	20
LD	RA1231	<5	<5	<5	<5	<5	20	40
	RA0669	<5	<5	<5	<5	160	80	5
	RA0778	<5	<5	<5	20	10	<5	<5
ctrl	RA1249	<5	ND	<5	<5	<5	<5	<5

C

IFN-ϒ ELISPOT

hIDUA peptide pools		PBMC					Spleen	Bone Marrow
		D0	D14	D30	D60	D90	D90	D90
HD IS	RA1528	-	-	-	-	-	-	-
	RA1404	-	-	-	-	-	-	-
	RA0747	-	-	-	-	+	-	-
HD	RA1329	-	+	+	+	-	-	-
	RA1280	-	-	+	+	-	-	-
	RA1219	-	+	+	+	-	-	-
LD	RA1231	+	+	+	+	-	-	-
	RA0669	-	-	-	+	+	+	-
	RA0778	-	+	+	+	+	+	+
ctrl	RA1249	-	+	-	-	-	-	-

AAV9 peptide pools		PBMC					Spleen	Bone Marrow
		D0	D14	D30	D60	D90	D90	D90
HD IS	RA1528	-	-	-	-	+	-	-
	RA1404	-	-	-	-	-	-	-
	RA0747	-	-	-	-	+	-	-
HD	RA1329	-	-	+	-	-	-	-
	RA1280	-	-	-	-	-	-	-
	RA1219	-	-	-	-	-	-	-
LD	RA1231	-	-	-	-	-	-	-
	RA0669	-	-	-	+	+	+	+
	RA0778	-	+	-	-	-	-	-
ctrl	RA1249	-	-	-	-	-	-	-

Figure 2. Immune Responses to the Transgene Product and Capsid in Rhesus Macaques Injected with Suboccipital AAV9.hIDUA

(A) Serum (left panel) and CSF (right panel) anti-hIDUA immune response measured by ELISA are shown. Results are shown as averages per group with SDs as error bars. Humoral responses were observed in the serum and CSF of all vector-treated animals and were not measurable in the HD IS animals at dilutions of 1:1,000 (serum) or 1:20 (CSF). (B) AAV9 NAb titers in serum (left panel) and CSF (right panel) are shown. (C). Interferon-gamma ELISPOT responses in PBMCs, spleen, and bone marrow toward hIDUA (left panel) and AAV9 capsid (right panel) peptide pools.

were similar or trended higher in the DRG from HD IS animals compared with HD animals. The vector was significantly distributed to the peripheral organs, especially the liver, with GCs around 1×10^6 GCs/μg DNA. We also recovered vector genomes from the lymphoid organs (spleen, lymph node, and bone marrow; 1×10^4 to 1×10^5 GCs/μg DNA). The eye, gonads, lungs, and thyroid exhibited the lowest vector distribution (1×10^2 to 1×10^3 GCs/μg DNA).

DISCUSSION

This study evaluated the safety of ICM administration of an AAV9 vector expressing hIDUA in nonhuman primates. Using a large-animal model and the intended clinical route of administration enabled us to assess toxicity related to both the vector and the injection procedure. We observed no adverse events associated with image-guided ICM injection and no histological lesions in vehicle-treated or day 14 vector-treated animals, supporting the safety of image-guided ICM injection.

Among vector-treated animals, we observed an unexpected toxicity consisting of asymptomatic degeneration of some sensory neuron cell bodies in the DRG and associated axons in the spinal cord and peripheral nerves. This toxicity was similar to the sensory ganglionitis that has been previously observed in piglets and juvenile nonhuman primates that were dosed intravenously with an AAV9 variant encoding human survival motor neuron 1; however, it had no clinical consequence, unlike what has been seen in piglets.[16] Interestingly, sensory neuron toxicity was absent in MPS I cats and MPS I dogs that were treated intrathecally with AAV9.fIDUA or AAV9.hIDUA, respectively, at doses similar to the nonhuman primate toxicology study.[12,14] At higher doses, non-tolerized MPS I dogs treated with AAV9.hIDUA (1×10^{13} GCs/kg, translating to about 10-fold higher dose than our nonhuman primates HD based on GCs/g of brain) developed a marked CSF pleocytosis, hindlimb weakness, and degeneration of lumbar motor neurons with infiltration of B and T lymphocytes; non-tolerized dogs treated with a similar dose to the ones used in our nonhuman primate studies (approximately 1×10^{11} GCs/g of brain) did not develop any neuronal or axonal degeneration.[14] To the best of our knowledge, no published reports exist of similar DRG toxicity in mice or rats dosed intrathecally with various AAV vectors. Rhesus macaques and piglets seem to be more sensitive to DRG toxicity. Hence, pigs or nonhuman primates would be useful models for assessing DRG toxicity with other intrathecal AAV programs. We do not know whether CSF pleocytosis is predictive of DRG toxicity because it did not strictly correlate with histological findings in the animals. Interestingly, CSF pleocytosis was observed as well in non-tolerized MPS I dogs treated with AAV9.hIDUA and was less pronounced in tolerized dogs.[14]

The mechanisms responsible for vector-induced sensory neuropathy are currently undefined. We observed that $CD3^+$ T cells were clustered around hIDUA-positive neurons, and we detected cellular immune responses against the hIDUA protein. However, these findings were still present in animals with pharmacologically suppressed T cell responses. Perhaps immune responses are not causative but worsen an initial injury; for instance, others have observed overexpression-induced neuronal toxicity in nonhuman primates after parenchymal injection of AAV expressing self-hexosaminidase

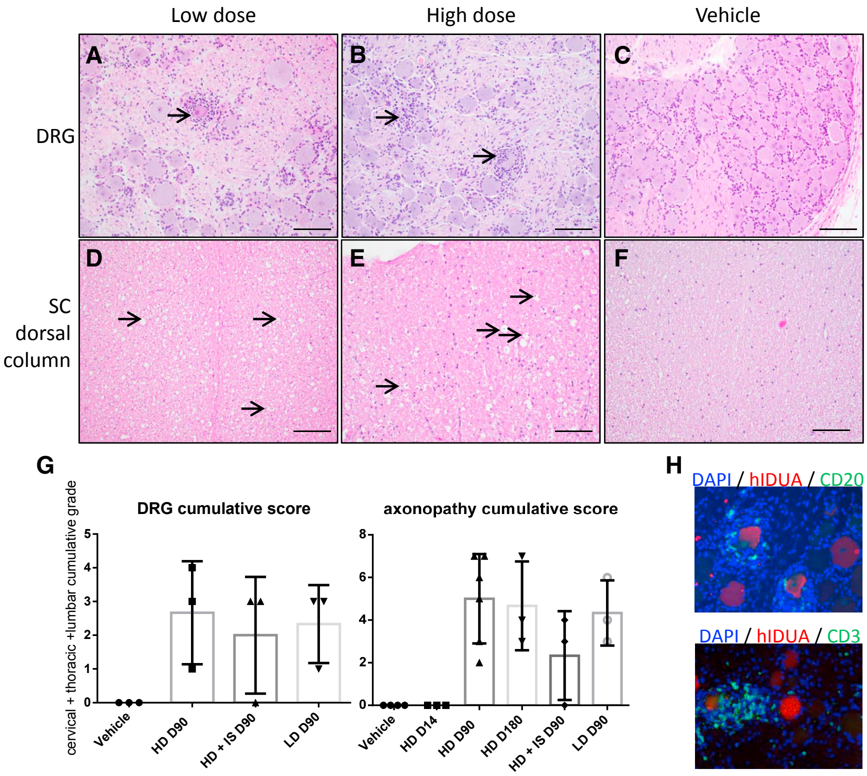

Figure 3. Representative CNS and PNS Histopathologic Findings in Rhesus Macaques Injected with Suboccipital AAV9.hIDUA

(A–C) The majority of animals had minimal to mild neuronal cell body degeneration in the DRG characterized by neurodegeneration (arrow, A), satellitosis, and mononuclear cell infiltrates that surrounded and invaded neuronal cell bodies (arrows, B). A normal DRG from a vehicle control animal is represented for comparison (C). (D–F) All animals from test article-treated groups had an axonopathy in the dorsal white matter tracts of the spinal cord that was bilateral and characterized by dilated myelin sheaths with and without myelomacrophages (arrows), consistent with axonal degeneration. An unremarkable section from the vehicle control group is shown for comparison (F). H&E staining was used. (G) Individual cumulative scores defined as the sum of cervical, thoracic, and lumbar segments scores with 1 as minimal, 2 as mild, 3 as moderate, 4 as marked, and 5 as severe; no animal had a score higher than 3 in any segment analyzed. Error bars represent SDs. (H) Immunofluorescence staining of the DRG using anti-hIDUA antibodies (red), anti-CD20 or anti-CD3 antibodies (green), and DAPI (nuclear counterstaining). Mononuclear cell infiltrates were predominantly composed of $CD3^+$ T cells (B) and $CD20^+$ B cells (C). Scale bars, 200 μm (A–C) and 100 μm (D–F).

under the control of a strong promoter similar to ours.[17] The immune response to the hIDUA protein is consistent with the relatively high immunogenicity of this protein and has been previously observed in murine, canine, and feline models of gene therapy, in addition to the high rate of antibody responses in MPS I patients treated with recombinant IDUA.[12–14,18] Irrespective of the mechanism of DRG toxicity, the findings suggest that these cells were heavily transduced following ICM AAV9 administration. Previous studies have reported efficient transduction of DRG utilizing systemic or intrathecal AAV delivery.[19–22] We currently do not know why sensory neurons are targeted using both routes of administration. DRG are outside of the blood-brain barrier and contain an area rich in neuronal cell bodies, which presents a high density of capillaries with a fenestrated endothelium.[23,24] The porous endothelium of DRG capillaries at least partially explains the robust transduction of these cells following systemic AAV administration. However, peripheral axon targeting followed by retrograde trafficking to the cell body could also occur, which would explain the high transduction of lower motor neurons following systemic AAV delivery.[25] Likewise, anatomic factors could explain efficient targeting of DRG neurons following intrathecal AAV delivery. The axons of DRG neurons are directly exposed to CSF in the dorsal roots, thus providing a means for axonal transduction by vector delivered into the CSF. Moreover, the spinal subarachnoid space is contiguous with the extracellular fluid of the DRG, which potentially allows the vector to pass directly from the CSF to the soma of DRG neurons.

The severe systemic toxicity that we observed recently in nonhuman primates following an intravenous delivery of high-dose AAV[16] was not detectable in these experiments despite the fact that the vector was substantially distributed outside of the CNS. This is not surprising given that the total dose of vector administered via ICM in this study is 50-fold lower than what we used in the intravenous studies. Instead, the toxicity we observed in this study was limited to minimal to moderate DRG neuron lesions. When comparing IS and non-IS vector-treated animals, our results suggest that IS can help alleviate the incidence and severity of the spinal cord and DRG test article-related findings. However, it is hard to draw any definitive conclusions due to study limitations, such as cohort size. We have shown in canine and feline models of MPS I that the rise of IDUA antibodies after delivering AAV9 gene therapy reduces but does not eliminate efficacy.[12,14] An additional benefit of IS is the modulation of adaptive immune responses to the transgene product, including T cells and antibodies against hIDUA, which should improve the therapeutic index.

In sum, this study of intrathecal AAV9-delivered hIDUA in rhesus macaques demonstrated safety of the suboccipital vector administration procedure, as well as an overall positive benefit-risk profile, for a devastating disorder such as MPS I, and specifically Hurler syndrome. Our findings support close monitoring for sensory neuron toxicity in human studies. It will be essential to frequently evaluate sensory symptoms such as paresthesias or changes in sensations of pain,

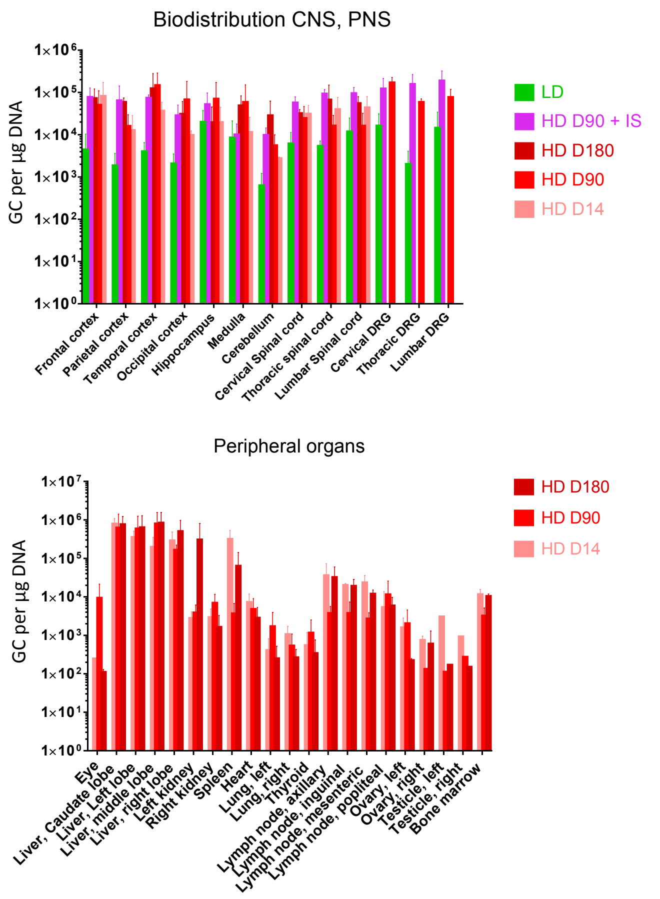

Figure 4. Biodistribution in Tissues from Rhesus Macaques Injected with Suboccipital AAV9.hIDUA

Vector GCs in the CNS and PNS (upper panel) and peripheral organs (lower panel) on selected time points after vector administration. Average results are shown with SDs as error bars.

temperature, touch, or proprioception. Quantitative sensory testing may enable more rigorous evaluation of subtle sensory changes. In addition, gathering objective measures, such as sensory nerve conduction, may be informative.

MATERIALS AND METHODS

Animals

All animal procedures were approved by the Institutional Animal Care and Use Committee of the University of Pennsylvania. Rhesus macaques (*Macaca mulatta*) that screened negative for AAV9 NAbs were procured from Covance Research Products (Princeton, NJ, USA). Animals were housed in an Association for Assessment and Accreditation of Laboratory Animal Care International-accredited Nonhuman Primate Research Program facility at the University of Pennsylvania in stainless-steel squeeze back cages. Animals received varied enrichments such as food treats, visual and auditory stimuli, manipulatives, and social interactions.

Study Design

This paper combines two studies; an overview of the study design and animals is presented in Table 1. We designed these studies to evaluate the safety, biodistribution, and pharmacology of two dose levels of the test article, a vector encoding a codon-optimized version of hIDUA for up to 180 days after administration via image-guided suboccipital puncture in rhesus macaques. The first study included 12 animals, and the second study included 10 animals, 3 of which were immunosuppressed via a combination of rapamycin and MMF. Both sexes were represented in all groups. Four macaques received the control article (artificial CSF) via suboccipital puncture. Eighteen rhesus macaques received the test article formulated in artificial CSF via suboccipital puncture. The animals were randomized to either receive an HD of 1×10^{13} GCs or an LD of 1×10^{12} GCs with or without IS, using an online randomized number generator (https://www.random.org). We collected blood and CSF as part of a general safety panel at time points indicated in Table 2. We also evaluated the humoral response and T cell responses to the transgene product hIDUA and the AAV9 capsid. After completing the in-life phase of the study, macaques were necropsied and tissues were harvested for further evaluation. We performed histopathologic evaluation and biodistribution of the vector genome in a comprehensive list of tissues from the CNS, peripheral nervous system (PNS), and peripheral organs as summarized in Table 3.

Test Article

The test article consisted of an AAV9 capsid packaging an expression construct with a hybrid promoter containing the cytomegalovirus enhancer with a chicken beta actin promoter (CB7), a chicken beta actin intron, a codon-optimized hIDUA transgene, and a rabbit beta-globulin polyadenylation signal. The expression construct was flanked by AAV serotype 2 inverted terminal repeats and was cloned in a plasmid containing a kanamycin resistance gene for manufacturing. The test article was manufactured under conditions as similar as possible to good manufacturing practice guidelines. The vector was produced by triple transfection of adherent HEK293 cells and purified from supernatant by affinity chromatography using a POROS CaptureSelect AAV9 resin (Thermo Fisher Scientific, Waltham, MA, USA), followed by anion exchange chromatography. Sterility of the test article was verified by direct immersion assay. Limulus amebocyte lysate and qPCR tests for endotoxin and mycoplasma, respectively, were negative. Vector titer by TaqMan PCR was 5.67×10^{13} GCs/mL. The purity of capsid proteins was 100%, as determined by SDS-PAGE analysis. Analytical ultracentrifugation indicated that the preparation contained 64% full vector particles. *In vitro* potency of the vector, assessed by IDUA enzyme expression, was confirmed to be similar to reference vector lots. The final product was diluted in Elliott's B Solution (Lukare Medical, Scotch Plain, NJ, USA) with 0.001% Pluronic F-68 (Thermo Fisher Scientific, Waltham, MA, USA). Dilutions were calculated using a standardized vector preparation form, and calculations were verified by designated personnel as indicated on the vector preparation forms. Unused vector preparations were archived and stored at −60°C to −80°C.

Table 3. Tissues Sampled at Necropsy

Tissues Collected for Histopathology		
Adrenal gland, left	large intestine, colon	pancreas
Adrenal gland, right	liver, caudate lobe	small intestine, duodenum
Aorta (thoracic and abdominal)	liver, left lobe	small intestine, jejunum
Ascending aorta (proximal)	liver, middle lobe	spinal cord
Bone marrow, femur	liver, right lobe	spleen
Brain, cerebellum	lung, left	stomach
Brain, cerebrum	lung, right	testicle, left
Dorsal root ganglia[a]	lymph node, axillary	testicle, right
Esophagus	lymph node, inguinal	thymus
Eyes (left)	lymph node, mesenteric	thyroid gland (with parathyroid)
Gall bladder	lymph node, popliteal	trachea
Heart	muscle, quadriceps femoris	trigeminal nerve ganglion[a]
Kidney, left	ovary, left	gross lesions (if any)
Kidney, right	ovary, right	injection site
Tissues Collected for Biodistribution		
Bone marrow, femur	kidney, right	ovary, left
Brain, cerebellum	liver, caudate lobe	ovary, right
Brain, frontal cortex	liver, left lobe	spinal cord, cervical
Brain, hippocampus	liver, middle lobe	spinal cord, lumbar
Brain, medulla	liver, right lobe	spinal cord, thoracic
Brain, occipital cortex	lung, left	spleen
Brain, parietal cortex	lung, right	testicle, left
Brain, temporal cortex	lymph node, axillary	testicle, right
Dorsal root ganglia[a]	lymph node, inguinal	thyroid gland (with parathyroid)
Eyes (right)	lymph node, mesenteric	trigeminal nerve ganglion[a]
Heart	lymph node, popliteal	gross lesions (if any)
Kidney, left	–	–
Tissues Collected for Lymphocyte Isolation		
Spleen	bone marrow	–

[a]DRG and trigeminal nerve ganglia were not sampled in the original study RGX151002p; RGX160822p was designed to further investigate CNS and PNS findings from RGX151002p. Therefore, all of the tissues listed here were sampled, but only the target CNS and PNS tissues were processed in the later study.

ICM Injection Procedure

Anesthetized macaques were transferred from animal holding to the procedure room and placed on an X-ray table in the lateral decubitus position with the head flexed forward for CSF collection and dosing ICM. The site of injection was aseptically prepared. Using aseptic technique, a 21G–27G, 1- to 1.5-inch Quincke spinal needle (Becton Dickinson, Franklin Lakes, NJ, USA) was advanced into the suboccipital space until the flow of CSF was observed, and 1 mL of CSF was collected for baseline analysis. The needle was directed at the wider superior portion of the cisterna magna to avoid potential brainstem injury. Correct placement of needle was verified by fluoroscopy (OEC 9800 C-arm; GE Healthcare, Little Chalfont, UK). After CSF collection, a Luer access extension catheter was connected to the spinal needle to facilitate dosing of 180 mg/mL Iohexol contrast media (GE Healthcare, Little Chalfont, UK). After verifying needle placement, a syringe containing the test article (volume equivalent to 1 mL plus the syringe volume and linker dead space) was connected to the flexible linker and injected over 30 ± 5 s. The needle was removed, and direct pressure was applied to the puncture site.

Verification of correct placement of the needle using a computed tomography scanner and contrast injection is key to mitigate the risk for accidental puncture or injection into the brainstem. This procedure, when translated to patients, will be performed in neurointerventional radiology suites and will use both angiography and intrathecal contrast injection to prevent accidental puncture of large vessels or brainstem.

Immunosuppression

A pilot study was conducted and demonstrated that orogastric gavage in chair-trained animals was a more reliable and reproducible method to achieve and maintain blood and plasma trough target levels when compared with treat-based voluntarily consumption. Animals were acclimated to the collar, pole capture, and chair restraint. Once acclimated, the animals were trained for insertion of an orogastric feeding tube and were then dosed through the feeding tube with a combination of commercially available IS drugs: a 200 mg/mL oral solution of MMF (CellCept; Roche Products) and 0.5, 1, or 2 mg coated tablets of rapamycin (Sirolimus, Rapamune; Greenstone) crushed in water. The IS regimen was initiated 3 weeks prior to intrathecal dosing of the test article. Rapamycin (0.75–2 mg/kg, once per day) and MMF (25–100 mg/kg, twice per day) dosages were adjusted to maintain a blood target trough level range of 2–3.5 μg/mL mycophenolic acid and 10–15 μg/L rapamycin. Rapamycin was used for the entirety of the study, whereas MMF was stopped on study day 60. Trough levels were monitored twice a week and IS doses were adjusted if the levels were either below or above the target range for two consecutive bleedings. Overall, rapamycin levels were steady and on target. MPA, the active metabolite of MMF, was below the target range of 2–3.5 μg/mL in RA1404 and had to be stopped earlier in this animal because of recurrent diarrhea (see Results).

Blood and CSF Analysis

Serum chemistry, hematology, coagulation, and CSF analyses were performed by the contract facility Antech GLP or Antech Diagnostics (Morrisville, NC, USA).

Immunology

Peripheral blood T cell responses against hIDUA and the AAV9 capsid were measured by interferon gamma (IFN-γ) enzyme-linked immunosorbent spot (ELISPOT) assays according to previously published methods,[26] using peptide libraries specific for AAV9 capsid and the hIDUA transgene. Positive response criteria were >55

spot-forming units per 10^6 lymphocytes and three times the medium negative control upon no stimulation. In addition, T cell responses were assayed in lymphocytes that were extracted from spleen and bone marrow after necropsy on study day 90. NAbs against AAV9 capsid were measured in serum and CSF using an *in vitro* HEK293 cell-based assay as previously described.[27] NAb titers are reported as the reciprocal of the sample dilution that inhibits transduction of 50% of the cells. The limit of detection of the assay was 1:5 sample dilution. Antibodies to hIDUA were measured in serum (1:1,000 sample dilution) and CSF (1:20 sample dilution) as previously described.[13]

Biodistribution

DNA was extracted from tissues using QIAamp Mini Extraction kits (Cat. 51306; Qiagen). Biodistribution analysis was performed by TaqMan qPCR targeting a vector polyadenylation signal sequence. Assays results were reported as GCs/μg DNA.

Necropsy

All animal procedures were approved by the Institutional Animal Care and Use Committee of the University of Pennsylvania. Animals were euthanized with intravenous pentobarbital overdose, necropsied, and tissues harvested for comprehensive histopathologic examination. Table 3 lists tissues that were collected and examined histologically. For study RGX160822p, only select tissues were examined by the study pathologist, which included CNS and PNS system tissues, as well as gross lesions.

Histology

Tissues were fixed in formalin, paraffin embedded, sectioned, and stained with H&E according to standard protocols. Tissues were evaluated histologically and peer-reviewed by two board-certified veterinary anatomic pathologists. The severity of lesions was graded as follows: grade 1 (minimal histopathologic change from inconspicuous to barely noticeable, affecting less than approximately 10% of the tissue); grade 2 (mild histopathologic change that is noticeable but not prominent, affecting approximately 10%–25% of the tissue); grade 3 (moderate histopathologic change that is prominent but not a dominant feature, affecting approximately 25%–50% of the tissue); grade 4 (marked histopathologic change that is dominant but not an overwhelming feature, affecting approximately 50%–95% of the tissue); grade 5 (severe histopathologic change that is an overwhelming feature, affecting greater than approximately 95% of the tissue). Three segments of the spinal cord (cervical, thoracic, lumbar) and at least three DRG from each segment (cervical, thoracic, lumbar) were evaluated. To ease global interpretation and allow comparison between groups, we also present combined scores for the spinal cord and DRG findings, representing the sum of the severity grades in cervical, thoracic, and lumbar segments with a range of 0–15.

For immunofluorescence, paraffin sections were deparaffinized through a series of xylene and ethanol treatments, boiled in a microwave for 6 minutes in 10 mM citrate buffer (pH 6.0), and treated with blocking buffer (1% donkey serum in PBS + 0.2% Triton for 10 min) followed by incubation with primary (1 hr) and fluorescence-labeled secondary (45 min; Jackson Immunoresearch, West Grove, PA, USA) antibodies diluted in blocking buffer. For dual staining, the two primary and two secondary antibodies were mixed together. Sections were mounted in Vectashield with DAPI (Vector Laboratories, Burlingame, CA, USA) to stain nuclei. Primary antibodies were rabbit polyclonal against CD3 (A045229-2; Agilent Technologies, Santa Clara, CA, USA) and CD20 (PA5-16701; Life Technologies, Carlsbad, CA, USA) and a sheep polyclonal against IDUA (AF4119; R&D Systems, Minneapolis, MN, USA).

SUPPLEMENTAL INFORMATION

Supplemental Information includes one figure and seven tables and can be found with this article online at https://doi.org/10.1016/j.omtm.2018.06.003.

AUTHOR CONTRIBUTIONS

J.H. and C.H. performed study design, investigation, data analysis, and writing; T.G. conducted animal dosing and veterinary care; N.K. participated in investigation; E.L.B. and L.K.R. performed the histopathology; P.B. supervised histology, immunohistochemistry, and immunofluorescence; R.C. conducted immune response analysis; L.K.R. supervised the study; and J.M.W. was in charge of conceptualization and supervision.

CONFLICTS OF INTEREST

J.M.W. is an advisor to, holds equity in, and has a sponsored research agreement with REGENXBIO; he also has a sponsored research agreement with Ultragenyx, Biogen, and Janssen, which are licensees of Penn technology. J.M.W. holds equity in Solid Bio and is an inventor on patents that have been licensed to various biopharmaceutical companies.

ACKNOWLEDGMENTS

This work was funded by REGENXBIO. We would like to thank Shu-Jen Chen and Michael Korn for biodistribution studies. Invaluable technical assistance was provided by the Nonhuman Primate Research Program, the Immunology Core, Vector Clinical Services, and the Morphology Core of the Gene Therapy Program of the University of Pennsylvania.

REFERENCES

1. Beck, M., Arn, P., Giugliani, R., Muenzer, J., Okuyama, T., Taylor, J., and Fallet, S. (2014). The natural history of MPS I: global perspectives from the MPS I Registry. Genet. Med. *16*, 759–765.
2. Vijay, S., and Wraith, J.E. (2005). Clinical presentation and follow-up of patients with the attenuated phenotype of mucopolysaccharidosis type I. Acta Paediatr. *94*, 872–877.
3. Matte, U., Yogalingam, G., Brooks, D., Leistner, S., Schwartz, I., Lima, L., Norato, D.Y., Brum, J.M., Beesley, C., Winchester, B., et al. (2003). Identification and characterization of 13 new mutations in mucopolysaccharidosis type I patients. Mol. Genet. Metab. *78*, 37–43.
4. Dahms, N.M., Lobel, P., and Kornfeld, S. (1989). Mannose 6-phosphate receptors and lysosomal enzyme targeting. J. Biol. Chem. *264*, 12115–12118.

5. Sando, G.N., and Neufeld, E.F. (1977). Recognition and receptor-mediated uptake of a lysosomal enzyme, α-l-iduronidase, by cultured human fibroblasts. Cell *12*, 619–627.

6. Clarke, L.A., Wraith, J.E., Beck, M., Kolodny, E.H., Pastores, G.M., Muenzer, J., Rapoport, D.M., Berger, K.I., Sidman, M., Kakkis, E.D., and Cox, G.F. (2009). Long-term efficacy and safety of laronidase in the treatment of mucopolysaccharidosis I. Pediatrics *123*, 229–240.

7. Sifuentes, M., Doroshow, R., Hoft, R., Mason, G., Walot, I., Diament, M., Okazaki, S., Huff, K., Cox, G.F., Swiedler, S.J., and Kakkis, E.D. (2007). A follow-up study of MPS I patients treated with laronidase enzyme replacement therapy for 6 years. Mol. Genet. Metab. *90*, 171–180.

8. Wraith, J.E., Clarke, L.A., Beck, M., Kolodny, E.H., Pastores, G.M., Muenzer, J., Rapoport, D.M., Berger, K.I., Swiedler, S.J., Kakkis, E.D., et al. (2004). Enzyme replacement therapy for mucopolysaccharidosis I: a randomized, double-blinded, placebo-controlled, multinational study of recombinant human α-L-iduronidase (laronidase). J. Pediatr. *144*, 581–588.

9. Wraith, J.E., Beck, M., Lane, R., van der Ploeg, A., Shapiro, E., Xue, Y., Kakkis, E.D., and Guffon, N. (2007). Enzyme replacement therapy in patients who have mucopolysaccharidosis I and are younger than 5 years: results of a multinational study of recombinant human alpha-L-iduronidase (laronidase). Pediatrics *120*, e37–e46.

10. de Ru, M.H., Boelens, J.J., Das, A.M., Jones, S.A., van der Lee, J.H., Mahlaoui, N., Mengel, E., Offringa, M., O'Meara, A., Parini, R., et al. (2011). Enzyme replacement therapy and/or hematopoietic stem cell transplantation at diagnosis in patients with mucopolysaccharidosis type I: results of a European consensus procedure. Orphanet J. Rare Dis. *6*, 55.

11. Kennedy, D.W., and Abkowitz, J.L. (1997). Kinetics of central nervous system microglial and macrophage engraftment: analysis using a transgenic bone marrow transplantation model. Blood *90*, 986–993.

12. Hinderer, C., Bell, P., Gurda, B.L., Wang, Q., Louboutin, J.P., Zhu, Y., Bagel, J., O'Donnell, P., Sikora, T., Ruane, T., et al. (2014). Intrathecal gene therapy corrects CNS pathology in a feline model of mucopolysaccharidosis I. Mol. Ther. *22*, 2018–2027.

13. Hinderer, C., Bell, P., Louboutin, J.P., Zhu, Y., Yu, H., Lin, G., Choa, R., Gurda, B.L., Bagel, J., O'Donnell, P., et al. (2015). Neonatal systemic AAV induces tolerance to CNS gene therapy in MPS I dogs and nonhuman primates. Mol. Ther. *23*, 1298–1307.

14. Hinderer, C., Bell, P., Louboutin, J.-P., Katz, N., Zhu, Y., Lin, G., Choa, R., Bagel, J., O'Donnell, P., Fitzgerald, C.A., et al. (2016). Neonatal tolerance induction enables accurate evaluation of gene therapy for MPS I in a canine model. Mol. Genet. Metab. *119*, 124–130.

15. Hinderer, C., Bell, P., Vite, C.H., Louboutin, J.-P., Grant, R., Bote, E., Yu, H., Pukenas, B., Hurst, R., and Wilson, J.M. (2014). Widespread gene transfer in the central nervous system of cynomolgus macaques following delivery of AAV9 into the cisterna magna. Mol. Ther. Methods Clin. Dev. *1*, 14051.

16. Hinderer, C., Katz, N., Buza, E.L., Dyer, C., Goode, T., Bell, P., Richman, L.K., and Wilson, J.M. (2018). Severe toxicity in honhuman primates and piglets following high-dose intravenous administration of an adeno-associated virus vector expressing human SMN. Hum. Gene Ther. *29*, 285–298.

17. Golebiowski, D., van der Bom, I.M.J., Kwon, C.S., Miller, A.D., Petrosky, K., Bradbury, A.M., Maitland, S., Kühn, A.L., Bishop, N., Curran, E., et al. (2017). Direct intracranial injection of AAVrh8 encoding monkey β-N-acetylhexosaminidase causes neurotoxicity in the primate brain. Hum. Gene Ther. *28*, 510–522.

18. Langereis, E.J., van Vlies, N., Church, H.J., Geskus, R.B., Hollak, C.E., Jones, S.A., Kulik, W., van Lenthe, H., Mercer, J., Schreider, L., et al. (2014). Biomarker responses correlate with antibody status in mucopolysaccharidosis type I patients on long-term enzyme replacement therapy. Mol. Genet. Metab. *114*, 129–137.

19. Gray, S.J., Nagabhushan Kalburgi, S., McCown, T.J., and Jude Samulski, R. (2013). Global CNS gene delivery and evasion of anti-AAV-neutralizing antibodies by intrathecal AAV administration in non-human primates. Gene Ther. *20*, 450–459.

20. Foust, K.D., Poirier, A., Pacak, C.A., Mandel, R.J., and Flotte, T.R. (2008). Neonatal intraperitoneal or intravenous injections of recombinant adeno-associated virus type 8 transduce dorsal root ganglia and lower motor neurons. Hum. Gene Ther. *19*, 61–70.

21. Jacques, S.J., Ahmed, Z., Forbes, A., Douglas, M.R., Vigenswara, V., Berry, M., and Logan, A. (2012). AAV8(gfp) preferentially targets large diameter dorsal root ganglion neurones after both intra-dorsal root ganglion and intrathecal injection. Mol. Cell. Neurosci. *49*, 464–474.

22. Zheng, H., Qiao, C., Wang, C.H., Li, J., Li, J., Yuan, Z., Zhang, C., and Xiao, X. (2010). Efficient retrograde transport of adeno-associated virus type 8 to spinal cord and dorsal root ganglion after vector delivery in muscle. Hum. Gene Ther. *21*, 87–97.

23. Godel, T., Pham, M., Heiland, S., Bendszus, M., and Bäumer, P. (2016). Human dorsal-root-ganglion perfusion measured in-vivo by MRI. Neuroimage *141*, 81–87.

24. Jimenez-Andrade, J.M., Herrera, M.B., Ghilardi, J.R., Vardanyan, M., Melemedjian, O.K., and Mantyh, P.W. (2008). Vascularization of the dorsal root ganglia and peripheral nerve of the mouse: implications for chemical-induced peripheral sensory neuropathies. Mol. Pain *4*, 10.

25. Bevan, A.K., Duque, S., Foust, K.D., Morales, P.R., Braun, L., Schmelzer, L., Chan, C.M., McCrate, M., Chicoine, L.G., Coley, B.D., et al. (2011). Systemic gene delivery in large species for targeting spinal cord, brain, and peripheral tissues for pediatric disorders. Mol. Ther. *19*, 1971–1980.

26. Gao, G., Wang, Q., Calcedo, R., Mays, L., Bell, P., Wang, L., Vandenberghe, L.H., Grant, R., Sanmiguel, J., Furth, E.E., and Wilson, J.M. (2009). Adeno-associated virus-mediated gene transfer to nonhuman primate liver can elicit destructive transgene-specific T cell responses. Hum. Gene Ther. *20*, 930–942.

27. Calcedo, R., Vandenberghe, L.H., Gao, G., Lin, J., and Wilson, J.M. (2009). Worldwide epidemiology of neutralizing antibodies to adeno-associated viruses. J. Infect. Dis. *199*, 381–390.

OMTM, Volume *10*

Supplemental Information

Toxicology Study of Intra-Cisterna Magna Adeno-Associated Virus 9 Expressing Human Alpha-L-Iduronidase in Rhesus Macaques

Juliette Hordeaux, Christian Hinderer, Tamara Goode, Nathan Katz, Elizabeth L. Buza, Peter Bell, Roberto Calcedo, Laura K. Richman, and James M. Wilson

Figure S1. Blood anomalies induced by the IS regimen. WBC counts, neutrophils, hemoglobin, ALT, phosphate, and albumin levels were transiently modified in at least one IS animal, after the onset of IS and before vector dosing, without any relation to intrathecal AAV9.hIDUA administration. Dotted lines represent the baseline average plus or minus two standard deviations. Black = vehicle control; red = HD; green = LD; purple = HD IS.

Figure S1.

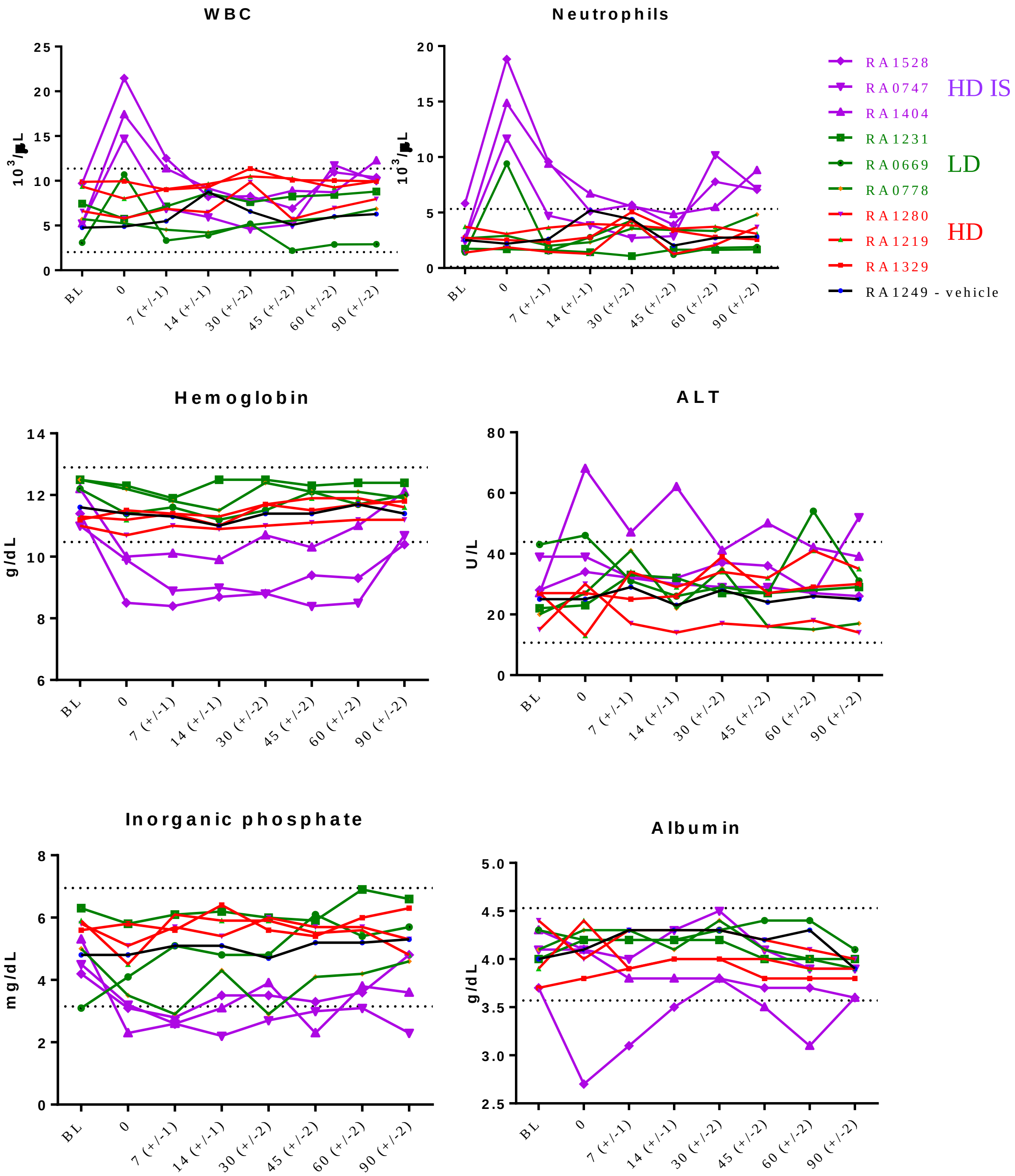
WBC
Neutrophils
Hemoglobin
ALT
Inorganic phosphate
Albumin
10^3/µL
g/dL
U/L
mg/dL
BL
0
7 (+/-1)
14 (+/-1)
30 (+/-2)
45 (+/-2)
60 (+/-2)
90 (+/-2)
RA1528
RA0747
RA1404
HD IS
RA1231
RA0669
RA0778
LD
RA1280
RA1219
RA1329
HD
RA1249 - vehicle

Table S1. Animal weights, study RGX150210p

Animal ID	Group		BL	D0	D3	D7 (+/-2)	D14 (+/-2)	D21 (+/-2)	D30 (+/-2)	D45 (+/-2)	D60 (+/-2)	D90 (+/-2)	D120 (+/-2)	D150 (+/-2)	D180 (+/-2)
RA1492	1A	HD D14	4.51	4.49	4.5	4.58	4.7	NA	NA	NA	NA	NA	NA	NA	NA
RA1292			5.49	5.54	5.66	5.48	5.51	NA	NA	NA	NA	NA	NA	NA	NA
RA0502			5.19	5.22	5.26	5.34	5.46	NA	NA	NA	NA	NA	NA	NA	NA
RA1470	1B	HD D90	4.93	5.05	5.05	4.92	5	5.27	5.6	5.91	5.75	5.9	NA	NA	NA
RA1514			4.41	4.33	4.39	4.4	4.64	4.41	4.39	4.86	4.95	4.65	NA	NA	NA
RA1287			5.36	5.44	5.49	5.66	5.55	5.71	5.6	5.55	5.7	5.75	NA	NA	NA
RA0773	1C	HD D180	5.65	5.75	5.79	5.98	6.08	6.19	6.45	6.51	6.3	6.5	6.25	5.85	6.05
RA1304			5.25	5.25	5.25	5.18	5.26	5.39	5.14	5.61	5.65	5.7	6.25	6.45	6.65
RA1532			5.39	5.74	5.86	5.8	5.9	6.07	6	5.7	6.05	6.45	6.35	6.05	6.1
RA1534	2	vehicle	5.09	5.15	5.09	5.2	5.4	NA	NA	NA	NA	NA	NA	NA	NA
RA0775			5.84	5.85	5.78	5.72	5.93	6.17	6.1	6.6	7.5	6.4	NA	NA	NA
RA1314			5.45	5.44	5.44	5.52	5.64	5.79	5.79	5.95	6	6.1	NA	NA	NA

Table S2. Animal weights, study RGX160822p

Animal ID	Group		BL	D0	D7 (+/- 1)	D14 (+/- 1)	D30	D45 (+/- 1)	D60 (+/- 2)	D90 (+/- 3)
RA1249	1	vehicle	6.60	6.55	6.50	6.60	6.80	6.75	6.90	7.30
RA1329	2	HD	8.85	8.75	8.85	9.00	9.10	8.50	9.00	9.20
RA1219			6.90	6.80	7.00	7.05	7.20	7.10	7.15	7.45
RA1280			7.80	7.90	7.85	8.00	8.30	8.60	8.75	9.25
RA0778	3	LD	4.10	4.10	4.05	4.10	4.10	4.20	4.30	4.25
RA0669			5.80	5.80	5.80	5.90	6.45	6.15	6.15	6.40
RA1231			8.05	7.80	7.75	7.85	7.85	8.10	8.20	8.70
RA1528	4	HD + IS	5.70	5.55	5.40	5.50	5.30	5.60	5.45	5.50
RA0747			5.85	5.75	5.65	5.80	5.75	5.70	5.70	5.85
RA1404			5.60	5.30	5.25	5.30	5.30	5.35	5.25	5.10

Table S3. CSF WBC counts

	D0	D3	D7	D14	D21	D30	D45	D60	D90	D120	D150	D180
RA1534 - vehicle	0	0	0	0	N/A	N/A	N/A	N/A	N/A	N/A	N/A	N/A
RA0775 - vehicle	0	0	0	0	0	0		0	0	N/A	N/A	N/A
RA1314 - vehicle	Blood	1	0	1	0	4	0	0	0	N/A	N/A	N/A
RA1249 - vehicle	0	N/A	0	Blood	Blood	0	1	1	0	N/A	N/A	N/A
RA0778 LD	0	N/A	Blood	0	N/A	1	1	0	0	N/A	N/A	N/A
RA0669 LD	0	N/A	0	0	N/A	0	7	10		N/A	N/A	N/A
RA1231 LD	0	N/A	0	0	N/A	2	1	2	0	N/A	N/A	N/A
RA1492 HD	0	0	0	0	N/A	N/A	N/A	N/A	N/A	N/A	N/A	N/A
RA1292 HD	4	1	2	0	N/A	N/A	N/A	N/A	N/A	N/A	N/A	N/A
RA0502 HD	0	1	0	0	N/A	N/A	N/A	N/A	N/A	N/A	N/A	N/A
RA1470 HD	Blood	2	0	17	12	Blood	28	14	4	N/A	N/A	N/A
RA1514 HD	Blood	3	0	0	12	11	4	1	1	N/A	N/A	N/A
RA1287 HD	0	0	0	0	10	27	2	1	0	N/A	N/A	N/A
RA1329 HD	0	N/A	0	0	N/A	0	0	4	2	N/A	N/A	N/A
RA1219 HD	0	N/A	0	0	N/A	*10.0**	1	2	0	N/A	N/A	N/A
RA1280 HD	0	N/A	0	0	N/A	3	2	2	0	N/A	N/A	N/A
RA0773 HD	0	0	0	0	4	4	11	1	0	1	0	0
RA1304 HD	0	1	3	0	1	1	0	2	1	3	0	2
RA1532 HD	0	1	0	6	18	27	11	3	7	16	13	Blood
RA1404 HD + IS	0	N/A	1	1	N/A	0	0	0	0	N/A	N/A	N/A
RA1528 HD + IS		N/A	0	0	N/A	0	0	0	0	N/A	N/A	N/A
RA0747 HD + IS	1	N/A	1	*10.00**	N/A	0	0	0	0	N/A	N/A	N/A

** values excluded: not confirmed by cytosmear analysis*

Blood: values excluded when red blood cell count > 500

Table S4. PBMC IFN-ϒ ELISPOT

			Average SFU per million PBMC							
**Group**	**Animal**	**Study Day**	**Medium**	AAV9-A	AAV9-B	AAV9-C	hIDUA-A	hIDUA-B	hIDUA-C	PHA + ctrl
Vehicle D14	RA1534	0	23	13	23	8	23	0	18	1490
		14	23	28	10	13	13	10	28	**1350**
HD D14	RA1492	0	20	20	18	13	23	15	15	**1330**
		14	25	13	23	10	5	20	15	**663**
	RA1292	0	23	33	13	18	15	8	15	**608**
		14	25	13	8	0	18	8	18	**515**
	RA0502	0	15	8	13	10	5	13	3	**570**
		14	15	18	8	8	15	13	5	**980**
LD D90	RA0778	0	8	28	20	53	40	23	25	**TNTC**
		14	18	43	**60**	**60**	**168**	**88**	53	**TNTC**
		30	25	25	23	23	**205**	NR	NR	**TNTC**
		60	80	65	110	105	**313**	233	**358**	**TNTC**
		90	28	33	50	28	**120**	**93**	**115**	**TNTC**
	RA0669	0	0	5	0	5	15	5	0	**1235**
		14	5	15	0	0	5	5	10	**TNTC**
		30	5	0	3	0	NR	NR	NR	**TNTC**
		60	13	18	**105**	**155**	**150**	**215**	**83**	**TNTC**
		90	0	15	23	**80**	45	**80**	10	**TNTC**
	RA1231	0	3	3	5	3	**63**	3	8	**TNTC**
		14	8	0	18	10	**85**	8	3	**TNTC**
		30	13	0	13	3	**120**	33	20	**TNTC**
		60	13	10	8	15	**113**	**128**	25	**TNTC**
		90	3	0	0	5	28	10	13	**TNTC**
HD D90	RA1470	0	5	5	5	0	8	5	23	**TNTC**
		21	5	8	5	15	8	3	3	**TNTC**
		90	3	15	40	8	**73**	8	10	**TNTC**
	RA1514	0	13	18	20	25	40	10	28	**TNTC**
		21	10	25	28	30	45	45	15	**TNTC**
		90	8	13	15	8	**100**	20	10	**1325**
	RA1287	0	5	10	8	5	5	30	10	**1185**
		21	5	8	10	25	53	25	8	**TNTC**
		90	5	15	13	20	50	**85**	8	**888**
	RA1329	0	0	3	13	0	3	8	3	**TNTC**
		14	3	35	20	5	**63**	23	15	**TNTC**
		30	3	**88**	3	25	**270**	**168**	48	**TNTC**
		60	25	18	35	8	**90**	53	23	**TNTC**
		90	8	20	5	0	43	20	18	**TNTC**
	RA1219	0	3	5	3	0	5	**108**	5	**TNTC**
		14	15	10	13	20	28	**138**	10	**TNTC**
		30	8	25	10	18	28	**73**	25	**TNTC**
		60	5	0	8	13	20	**58**	43	**TNTC**
		90	3	0	0	0	3	5	3	**TNTC**
	RA1280	0	13	15	20	10	30	10	13	**TNTC**
		14	20	8	20	10	48	5	13	**TNTC**
		30	25	10	20	25	**78**	45	35	**TNTC**
		60	20	5	20	18	**85**	35	60	**TNTC**
		90	3	18	10	20	55	15	23	**TNTC**
HD + IS D90	RA1404	0	5	10	5	5	5	0	35	**TNTC**
		30	0	0	0	0	3	3	3	**TNTC**
		60	0	0	3	3	0	5	5	**TNTC**
		90	35	13	20	40	28	25	20	**TNTC**
	RA1528	0	0	0	13	15	13	8	0	**TNTC**
		30	8	5	13	10	8	5	0	**TNTC**
		60	8	13	28	35	5	18	13	**TNTC**
		90	13	38	40	**85**	28	25	30	**TNTC**
	RA0747	0	5	13	18	8	18	15	8	**TNTC**
		30	0	13	10	8	20	0	5	**TNTC**
		60	20	23	33	38	13	20	0	**TNTC**
		90	23	33	68	**160**	**110**	**95**	38	**TNTC**

Table S4. (cont.) PBMC IFN-ϒ ELISPOT

Group	Animal #	Study Day	Medium	AAV9 A	AAV9 B	AAV9 C	hIDUA A	hIDUA B	hIDUA C	PHA + ctrl
			Average SFU per million PBMC							
Vehicle D90	RA0775	0	20	13	55	35	40	38	38	**TNTC**
		21	13	13	25	25	10	15	20	**TNTC**
		90	23	30	30	35	20	30	10	**TNTC**
	RA1314	0	23	13	13	5	18	18	10	**1340**
		21	13	3	10	10	0	15	3	**1390**
		90	3	18	13	13	33	10	20	**TNTC**
	RA1249	0	15	20	0	5	5	5	0	**1425**
		14	28	8	13	35	18	25	**125**	**TNTC**
		30	28	43	25	NR	25	20	NR	**TNTC**
		60	53	63	33	20	28	30	35	**TNTC**
		90	8	8	3	10	10	0	18	**TNTC**
HD D180	RA0773	0	5	15	3	10	10	5	15	**1520**
		21	3	8	3	10	13	10	0	**1560**
		90	8	0	8	5	10	15	8	**900**
		180	30	15	20	33	45	45	20	**2000**
	RA1304	0	8	0	0	0	0	0	0	**265**
		21	5	3	3	0	5	3	5	**95**
		90	3	3	8	8	0	13	8	**253**
		180	15	18	18	28	28	20	13	**503**
	RA1532	0	5	3	0	5	8	0	3	**TNTC**
		21	0	3	0	3	8	8	8	**TNTC**
		90	23	23	13	30	**70**	55	20	**TNTC**
		180	18	**68**	**65**	**108**	**95**	**63**	**65**	**TNTC**

SFU = Spot Forming Unit. NR = not enough cells

Positive responses, defined as an average value >55 SFU/million PBMCs and at least three times greater than the medium negative control, are highlighted in red. TNTC = too numerous to be counted

Table S5. Incidence and severity of the DRG finding

Study	RGX151002p					RGX160822p			
Necropsy Day	14		90		180	90			
Group	1A	2A	1B	2B	1C	1	2	3	4
Dose (GC)	10^{13}	Control	10^{13}	Control	10^{13}	Control	10^{13}	10^{12}	10^{13}
Immunosuppression	No	No	No	No	No	No	No	No	Yes
Number of Animals	3	1	3	2	3	1	3	3	3
No. examined	0	0	0	0	0	1	3	3	3
**Dorsal root ganglion, Cervical**									
Neuronal cell body degeneration with mononuclear cell infiltrate									
No significant abnormalities	-	-	-	-	-	1 (M)	1	1	1
Grade 1	-	-	-	-	-	-	2	2	2
**Dorsal root ganglion, Thoracic**									
Neuronal cell body degeneration with mononuclear cell infiltrate									
No significant abnormalities	-	-	-	-	-	1	0	1	1
Grade 1	-	-	-	-	-	-	3	2	2
**Dorsal root ganglion, Lumbar**									
Neuronal cell body degeneration with mononuclear cell infiltrate									
No significant abnormalities	-	-	-	-	-	1	0	0	2
Grade 1	-	-	-	-	-	-	3	3	-
Grade 2	-	-	-	-	-	-	-	-	1

M=missing tissue

The findings in the DRG consisted of neuronal degeneration characterized by central chromatolysis, satellitosis, and mononuclear cell infiltrates that surrounded and invaded neuronal cell bodies (neuronophagia). DRG from the initial study, RGX151002p, were not sampled.

Table S6. Incidence and severity of the axonopathy finding

**Study**	**RGX151002p**					**RGX160822p**			
**Necropsy Day**	**14**		**90**		**180**	**90**			
**Group**	**1A**	**2A**	**1B**	**2B**	**1C**	**1**	**2**	**3**	**4**
**Dose (GC)**	**10^{13}**	**Control**	**10^{13}**	**Control**	**10^{13}**	**Control**	**10^{13}**	**10^{12}**	**10^{13}**
**Immunosuppression**	**No**	**No**	**No**	**No**	**No**	**No**	**No**	**No**	**Yes**
**Number of Animals**	**3**	**1**	**3**	**2**	**3**	**1**	**3**	**3**	**3**
No. examined	3	1	3	2	3	1	3	3	3
**Spinal Cord, Cervical**									
Axonopathy, dorsal white matter tracts									
No significant abnormalities	3	1	0	2	0	1	1	0	2
Grade 1	-	-	-	-	2	-	-	2	1
Grade 2	-	-	2	-	1	-	2	1	-
Grade 3	-	-	1	-	-	-	-	-	-
**Spinal Cord, Thoracic**									
Axonopathy, dorsal white matter tracts									
No significant abnormalities	3	1	0	2	0	1	0	0	1
Grade 1	-	-	2	-	2	-	2	3	2
Grade 2	-	-	1	-	1	-	1	-	-
**Spinal Cord, Lumbar**									
Axonopathy, dorsal white matter tracts									
No significant abnormalities	3	1	0	2	0	1	0	0	1
Grade 1	-	-	-	-	1	-	2	1	1
Grade 2	-	-	3	-	2	-	1	2	1

The dorsal white matter tracts of the spinal cord had an axonopathy characterized by dilated myelin sheaths with and without myelomacrophages, which was consistent with axonal degeneration.

Table S7. Biodistribution (vector GCs) in tissues

	HD D14			HD D90						HD D180			HD D90 + IS			LD		
	RA0502	RA1292	RA1492	RA1287	RA1470	RA1514	RA1219	RA1280	RA1329	RA0773	RA1304	RA1532	RA0747	RA1404	RA1528	RA0669	RA0778	RA1231
Frontal cortex	185000	33500	17600	17300	78500	9920	160000	24800	530	124000	61800	23500	48700	135000	40600	1770	11100	<50
Parietal cortex	30600	2700	3380	24700	33500	2780	26500	4660	595	44300	76000	50000	10800	154000	21800	181	3720	1520
Temporal cortex	29500	60600	16000	52200	215000	14900	368000	201000	137	34000	302000	23400	52700	78300	82600	2650	6900	2100
Occipital cortex	12200	9440	6490	34100	303000	6780	22500	23700	536	64500	4530	19900	21100	52600	8630	3280	2380	353
Hippocampus	3370	48400	4940	4110	94700	18100	23700	267000	97.6	6050	1400	49000	103000	13600	33800	15500	39200	3090
Medulla	5360	239	27700	32900	14100	19000	25000	250000	438	9170	82000	49900	18800	2800	7280	544	23200	846
Cerebellum	2390	2950	2660	300	8740	11800	6350	4690	368	67700	7960	6370	12900	11800	3550	387	1290	145
Cervical Spinal cord	39100	42800	7830	22200	35100	5370	62800	15700	561	28800	39900	23500	28400	61900	74300	5330	11500	1070
Thoracic spinal cord	80500	20500	14000	9460	2950	26900	22200	31800	785	22300	161000	11100	120000	77100	72200	7320	3890	4370
Lumbar Spinal cord	81400	39400	6590	4950	3080	12100	37800	34800	851	83700	38300	37400	79800	59900	135000	4970	26800	2490
Cervical DRG	N/A	N/A	N/A	N/A	N/A	N/A	208000	121000	N/A	N/A	N/A	N/A	209000	19600	125000	12600	32400	1990
Thoracic DRG	N/A	N/A	N/A	N/A	N/A	N/A	66600	47300	N/A	N/A	N/A	N/A	103000	66600	282000	1260	4310	232
Lumbar DRG	N/A	N/A	N/A	N/A	N/A	N/A	105000	43600	N/A	N/A	N/A	N/A	81800	121000	345000	3260	37300	1200
Eye	<50	int	237	301	17700	<50	N/A	N/A	N/A	105	130	82	N/A	N/A	N/A	N/A	N/A	N/A
Liver, Caudate lobe	415000	1070000	784000	1530000	181000	91100	N/A	N/A	N/A	1210000	763000	210000	N/A	N/A	N/A	N/A	N/A	N/A
Liver, Left lobe	307000	190000	519000	1350000	189000	167000	N/A	N/A	N/A	1380000	334000	110000	N/A	N/A	N/A	N/A	N/A	N/A
Liver, middle lobe	361000	159000	45300	1680000	324000	290000	N/A	N/A	N/A	1680000	268000	449000	N/A	N/A	N/A	N/A	N/A	N/A
Liver, right lobe	499000	250000	86900	135000	228000	114000	N/A	N/A	N/A	1040000	309000	110000	N/A	N/A	N/A	N/A	N/A	N/A
Left kidney	2570	1270	4130	5210	5000	945	N/A	N/A	N/A	882000	1580	430	N/A	N/A	N/A	N/A	N/A	N/A
Right kidney	3300	613	4510	9700	9280	870	N/A	N/A	N/A	775	3530	380	N/A	N/A	N/A	N/A	N/A	N/A
Spleen	536000	78200	301000	1360	7120	2140	N/A	N/A	N/A	154000	5860	23000	N/A	N/A	N/A	N/A	N/A	N/A
Heart	7430	11500	1820	1970	9670	2050	N/A	N/A	N/A	2400	5420	280	N/A	N/A	N/A	N/A	N/A	N/A
Lung, left	113	181	878	197	4270	438	N/A	N/A	N/A	145	556	int	N/A	N/A	N/A	N/A	N/A	N/A
Lung, right	211	1390	1440	148	1160	227	N/A	N/A	N/A	54.6	371	341	N/A	N/A	N/A	N/A	N/A	N/A
Thyroid	int	54.8	991	417	180	2750	N/A	N/A	N/A	831	84.9	63.3	N/A	N/A	N/A	N/A	N/A	N/A
Lymph node, axillary	61300	7550	int	5090	1310	4330	N/A	N/A	N/A	17200	11500	64500	N/A	N/A	N/A	N/A	N/A	N/A
Lymph node, inguinal	19500	16300	22200	7840	1450	1470	N/A	N/A	N/A	29700	13400	12000	N/A	N/A	N/A	N/A	N/A	N/A
Lymph node, mesenteri	12100	37400	18300	1590	2150	4030	N/A	N/A	N/A	9920	15500	9010	N/A	N/A	N/A	N/A	N/A	N/A
Lymph node, popliteal	113	15000	200	5540	27600	64.8	N/A	N/A	N/A	8650	1150	7100	N/A	N/A	N/A	N/A	N/A	N/A
Ovary, left	625	N/A	2440	N/A	3770	121	N/A	N/A	N/A	237	N/A	190	N/A	N/A	N/A	N/A	N/A	N/A
Ovary, right	555	N/A	881	N/A	127		N/A	N/A	N/A	1080	N/A	72.2	N/A	N/A	N/A	N/A	N/A	N/A
Testicle, left	N/A	2950	N/A	107	N/A	N/A	N/A	N/A	N/A	N/A	163	N/A	N/A	N/A	N/A	N/A	N/A	N/A
Testicle, right	N/A	888	N/A	262	N/A	N/A	N/A	N/A	N/A	N/A	144	N/A	N/A	N/A	N/A	N/A	N/A	N/A
Bone marrow	6270	14700	12500	734	4080	4410	N/A	N/A	N/A	11500	10600	7760	N/A	N/A	N/A	N/A	N/A	N/A
N/A Not Applicable																		
int. interference																		
